# Sunitinib induces immunogenic cell death through eIF2α phosphorylation to potentiate immunotherapy in HCC

**DOI:** 10.1016/j.isci.2026.115378

**Published:** 2026-03-16

**Authors:** Yimei Gu, Hanbing Mai, Peijian Huang, Hui Yuan, Mingxuan Sun, Huixin Gao, Chenyu Shang, Yangfen Ou, Ting Liu, Xianzhang Huang, Jizhou Tan

**Affiliations:** 1 The Second Clinical Medical College, Guangzhou University of Chinese Medicine, Clinical Laboratory/State Key Laboratory of Traditional Chinese Medicine Syndrome, Guangdong Provincial Hospital of Chinese Medicine, Guangzhou 510120, China; 2 The First Affiliated Hospital, Sun Yat-sen University, Guangzhou 510080, China; 3 State Key Laboratory Dampness Syndrome of Chinese Medicine, Guangzhou 510120, China; 4 Department of Clinical Laboratory, Guangzhou Women and Children Medical Center, Guangzhou Medical University, Guangzhou, Guangdong 510600, China; 5 Department of Laboratory Medicine, Baiyun District People’s Hospital of Guangzhou, Guangzhou, Guangdong 510500, China

**Keywords:** immunology, pharmacology, molecular biology, cancer systems biology, cancer

## Abstract

Hepatocellular carcinoma (HCC) prognosis remains poor due to its immunosuppressive tumor microenvironment. Inducing immunogenic cell death (ICD) represents a promising strategy to remodel this environment. Herein, we developed an ICD-related risk signature (ICDRS) to stratify patients and predict immunotherapy response. Through integrated database screening, sunitinib was repurposed as an ICD inducer. It elicited hallmark features of ICD, including calreticulin exposure, ATP release, and HMGB1 secretion, and effectively suppressed organoid growth. Sunitinib-pretreated HCC cells promoted dendritic cell (DC) maturation *in vitro*, and these pretreated cells protected mice from tumor rechallenge as a vaccine. Mechanistically, sunitinib induced ICD via eIF2α phosphorylation, as confirmed by reversal with the inhibitor ISRIB. Moreover, sunitinib synergized with anti-PD-L1 therapy by enhancing DC activation and increasing intratumoral cytotoxic T cells, while reducing immunosuppressive cell populations, resulting in potent tumor control. Together, our work provides a clinically actionable strategy to personalize and enhance immunotherapy outcomes in HCC.

## Highlights

• Repurposing sunitinib as a novel immunogenic cell death (ICD) inducer for HCC
• An ICD-related risk signature predicts patient response to immunotherapy
• Sunitinib synergizes with anti-PD-L1 by enhancing DC maturation via ICD

## Graphical abstract

**Figure:**
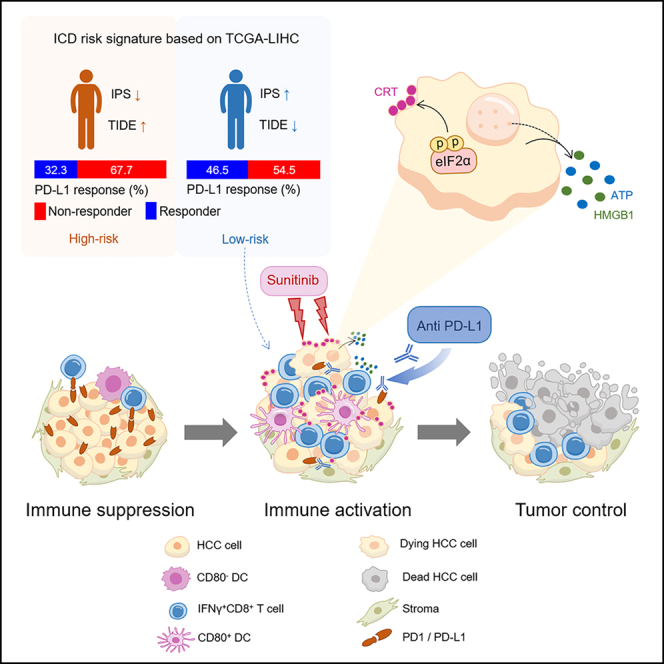

## Introduction

Hepatocellular carcinoma (HCC), the most prevalent form of liver cancer, ranks as the sixth most diagnosed malignancy globally and the third leading cause of cancer-related mortality.^1^ Despite therapeutic advances—including tyrosine kinase inhibitors (TKIs) and immune checkpoint inhibitors (ICIs)—the five-year survival rate for advanced HCC remains below 20%, driven by intrinsic therapeutic challenges and an immunosuppressive tumor microenvironment (TME) that facilitates immune evasion.^2^^,^^3^ These limitations underscore the urgent need for strategies that rewire the TME to activate sustained anti-tumor immunity.

Immunogenic cell death (ICD), a regulated cell death modality triggered by maladaptive stress responses, has emerged as a pivotal mechanism to convert immunologically “cold” tumors into “hot” niches.^4^^,^^5^^,^^6^ ICD is characterized by two sequential hallmarks: (1) phosphorylation of eukaryotic initiation factor 2α (*p*-eIF2α), which drives calreticulin (CRT) surface exposure via the integrated stress response (ISR), and (2) autophagy-mediated ATP secretion. These damage-associated molecular patterns (DAMPs) engage dendritic cells (DCs) and cytotoxic T lymphocytes (CTLs) to orchestrate tumor-specific adaptive immunity.^7^^,^^8^ Critically, ICD induction sensitizes tumors to ICIs by enhancing antigen presentation and immune infiltration.^9^^,^^10^^,^^11^^,^^12^ Consequently, specific expression patterns of ICD-related gene (ICDRG) may have important prognostic value in predicting ICI response, as they reflect the ability of the TME to sustain immunogenic signals.^13^

Current ICD inducers for HCC—including doxorubicin, oxaliplatin, and certain radiotherapy modalities—face limitations such as systemic toxicity and heterogeneous therapeutic responses.^14^^,^^15^ To expand the repertoire of clinically translatable ICD inducers, we aimed to identify FDA-approved drugs capable of inducing HCC-specific ICD, leveraging their established safety profiles and repurposing potential as a cost-effective alternative to *de novo* drug development. Through screening of ICD-related differential expression genes (DEGs) in the TCGA-LIHC cohort, followed by cross-database screening using three independent pharmacological platforms: CMap, L1000 FWD, and DGIdb, we identified sunitinib as a potential ICD inducer in HCC. Sunitinib inhibits angiogenesis via VEGFR/PDGFR blockade and directly suppresses tumor growth through kinase inhibition.^16^ Notably, although a phase III trial demonstrated that sunitinib did not provide a superior overall survival benefit to sorafenib in patients with HCC, accumulating evidence suggests that sunitinib, unlike sorafenib, may overcome tumor-induced immunotolerance by suppressing PD-1 expression and enhancing T cell infiltration, thereby activating anti-tumor immunity.^7^^,^^17^^,^^18^^,^^19^ While these studies highlight its immunomodulatory potential, the precise mechanism, particularly its capacity to induce ICD—a distinct process characterized by the emission of DAMPs that primes adaptive immunity—remains unexplored in HCC.

In our study, sunitinib rapidly enhanced the phosphorylation level of eIF2α in HCC cells, driving CRT translocation and ATP/HMGB1 release. Importantly, sunitinib-primed tumor cells promoted dendritic cell maturation *in vitro* and provided significant protection against secondary tumor implantation as vaccines in immunocompetent mice. In murine models, sunitinib promoted IFN-γ^+^ CD8^+^ T cells infiltration and reduced Tregs accumulation in the TME. Furthermore, it synergized with PD-L1 blockade to suppress tumor growth. These findings provide preclinical rationale for combining eIF2α-targeted ICD inducers with ICIs in HCC, addressing a critical unmet need in oncology.

## Results

### Construction and validation of ICD related risk signature in HCC

**Figure 1:**
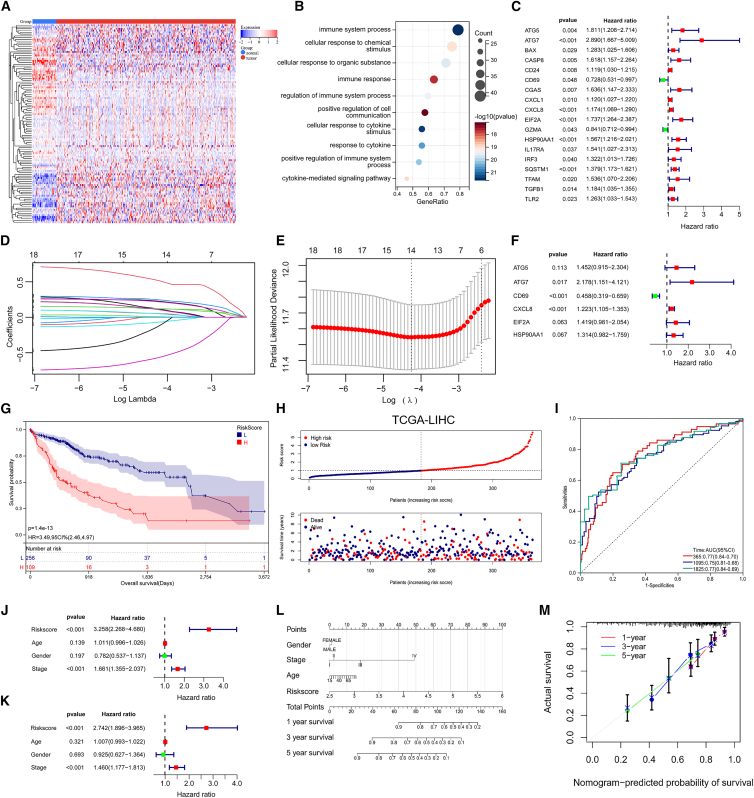
Construction and validation of ICD related risk signature in HCC (A) Heatmaps illustrating the expression of 112 prognostic ICDRGs in TCGA-LIHC cohort. (B) GO enrichment analysis of ICD-associated DEGs. (C) Univariate Cox analysis identified 18 genes associated with survival (*p* value <0.05). (D) 10-fold cross-validation calculates the confidence index under each lambda. (E) The variation of each characteristic coefficient under different lambda, where the horizontal axis represents the log value of the independent variable lambda, and the vertical axis represents the coefficient of the independent variable. (F) Forest plots of the results of the multivariate cox analysis of 6 prognostic ICDRGs in HCC. (G) Kaplan-Meier curves of patients in the high- and low-risk groups based on the 6 ICDRGs signature in TCGA-LIHC. (H) The distribution of risk scores and survival time of patients in TCGA-LIHC. (I) The AUCs of the ROC curves for 1-, 3-, and 5-year OS prediction in TCGA-LIHC. (J and K) Forest plots of the independent factors established by incorporating the risk scores and clinicopathologic characteristics. (L) Nomogram based on risk score, age, stage and gender to predict 1-, 3-, and 5-year OS. (M) Calibration curves of nomogram for 1-, 3-, and 5-year OS.

To establish the ICD related risk signature (ICDRS), we first analyzed the expression landscape of 112 ICDRGs in the TCGA-LIHC cohort (Figure 1A). Through comparative transcriptomic profiling of HCC specimens versus matched normal tissues (screening threshold: *p* < 0.01, |fold change| > 1.2), we identified 52 differentially expressed genes (DEGs) with significant alterations (Table S1). Gene ontology (GO) enrichment analysis demonstrated pronounced association of these DEGs with core immunological functions, particularly in immune system processes (GO:0002376, *p* < 0.01) and cellular response to chemical stimulus (GO: 0070887, *p* < 0.01) (Figure 1B). We subsequently performed survival-based prioritization using univariate Cox proportional hazards regression, identifying 18 ICDRGs with prognostic significance (*p* < 0.05) (Figure 1C). We then employed LASSO regression with 10-fold cross-validation (optimal λ selected by minimum deviance; Figures 1D and 1E), followed by multivariate Cox regression. This process identified six pivotal ICDRGs (Figure 1F), which were incorporated into the risk stratification model. The prognostic risk score was computed using the formula: risk score = (0.282) × *ATG5* + (0.658) × *ATG7* + (−0.720) × *CD69* + (0.130) × *CXCL8* + (0.299) × *EIF2A* + (0.272) × *HSP90AA1*, where coefficients represent multivariate Cox regression weights. Applying this algorithm to the complete TCGA-LIHC cohort (*n* = 371) stratified HCC patients into distinct low- and high-risk subgroups (median cutoff). In this cohort, patients stratified by the ICDRS risk score showed significant differences in overall survival (OS), with high-risk patients exhibiting markedly poorer outcomes compared to the low-risk group (HR = 3.49, 95% CI = 2.46–4.97, *p* < 0.001; Figure 1G). Consistent with the TCGA-LIHC findings, both validation cohorts also demonstrated a strong association between high ICDRS risk and reduced OS (Figures S1A and S1D). Moreover, the trends in the distribution of risk scores and survival times were consistent across the three cohorts mentioned previously, indicating the stability of the ICDRS model (Figures 1H, S1B, and S1E). Time-dependent ROC analysis further confirmed the prognostic accuracy of the model. In the TCGA-LIHC cohort (development cohort), the AUC values for 1-, 3-, and 5-year survival predictions were consistently robust, exceeding 0.70 at all timepoints (Figure 1I). In the external validation cohorts, the model demonstrated modest yet consistent discriminative ability across both the GSE14520 and LIRI-JP datasets (Figures S1C and S1F).

Univariate and multivariate Cox regression adjusting for clinicopathological confounders (age, gender, and TNM stage) established the ICDRS as an independent prognostic determinant (HR = 3.258, 95% CI:2.268–4.680, *p* < 0.001; HR = 2.742, 95% CI:1.896–3.965, *p* < 0.001; Figures 1J and 1K). To facilitate clinical translation, we developed a dynamic nomogram integrating ICDRS with key clinical parameters (age, gender, and stage; Figure 1L). The model demonstrated excellent calibration accuracy and discrimination for 1–5 years survival prediction (Figure 1M).

Collectively, we constructed and validated a prognostic signature based on six ICD-related genes (*ATG5*, *ATG7*, *CD69*, *CXCL8*, *EIF2A*, and *HSP90AA1*) that effectively stratifies HCC patients into distinct risk groups, providing a reliable tool for prognostic assessment and precision oncology.

### Analysis of HCC immune microenvironment heterogeneity in the ICDRS model and screening for ICD inducers

**Figure 2:**
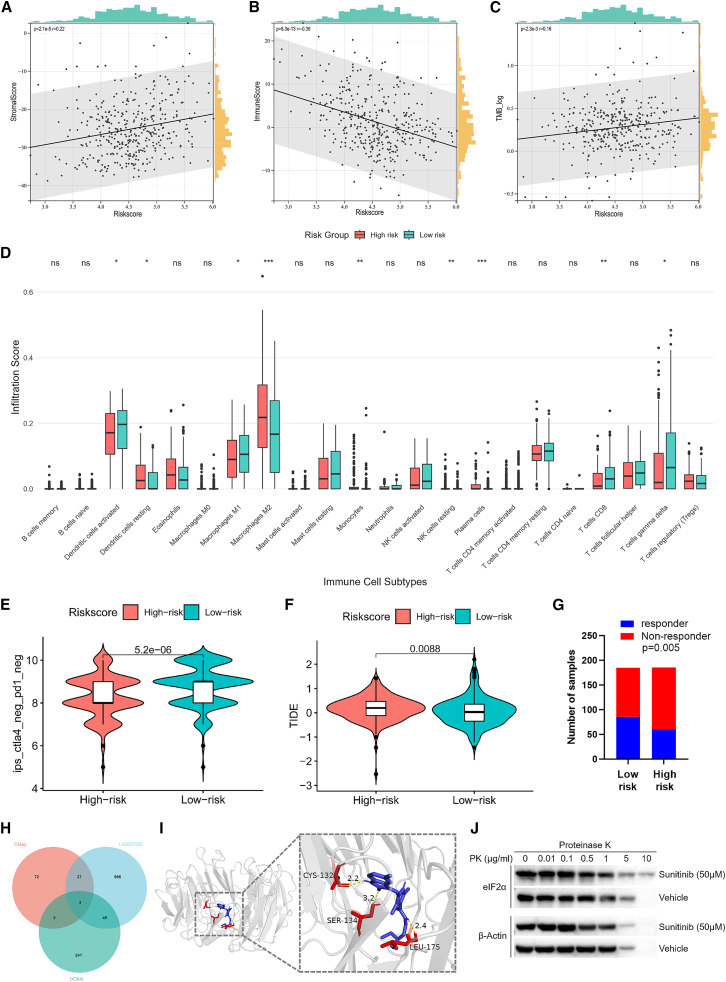
Immune characteristics of the ICDRS model and repurposed drug discovery in HCC (A and B) Correlation of risk score in TCGA-LIHC with stromal, immune according to the ESTIMATE algorithm. (C) Correlation analysis between risk score and TMB. (D) Comparison of immune cell infiltration levels between ICDRS subtypes of TCGA-LIHC. *ns*, not significant; ∗, *p* < 0.05; ∗∗, *p* < 0.01; ∗∗∗, *p* < 0.001. (E) The relative distribution of IPS identified by the status of CTLA-4 and PD-1 was compared between high-versus low-risk group in TCGA-LIHC cohort. (F) Correlation of TIDE status and ICD related risk signature in HCC patients. (G) Predictive results of TIDE algorithm on anti-PD-L1 response rates in HCC patients from TCGA-LIHC. (H) Drug candidates identified via cross-analysis of the CMap, L1000 FWD, and DGIdb databases were sunitinib, quercetin, and erythromycin. (I) Molecular docking of eIF2α (PDB ID: 8DYS) with sunitinib showing the interaction site. (J) DARTs was performed in untreated Hepa1-6 cells lysates incubated with sunitinib at 50 μM.

To delineate the immunological heterogeneity of ICDRS model, we systematically deconvoluted the TME in the TCGA-LIHC cohort using ESTIMATE and CIBERSORT algorithms. Correlation analysis showed that Stromal Score was significantly positively correlated with risk score (*p* < 0.001, r = 0.22; Figure 2A), indicating that microenvironmental fibrosis was lower in patients with a low score (low-risk patients). In contrast, patients were more immunoreactive when their risk score was lower (*p* < 0.001, r = −0.36; Figure 2B). Notably, despite a positive correlation between tumor mutational burden (TMB) and risk score (*p* < 0.01, r = 0.16; Figure 2C), patients in the low-risk group exhibited preferential enrichment of anti-tumor immune cells, including CD8^+^ T cells (*p* < 0.01), γδ T cells (*p* < 0.05), and activated DCs (*p* < 0.05), along with a reduction in immunosuppressive M2 macrophages (*p* < 0.001; Figure 2D). Further validation through the immunophenoscore (IPS) framework highlighted differential immunotherapeutic potential across risk groups. Low-risk patients exhibited significantly higher IPS scores identified by the four CTLA-4 or PD-1 statuses, suggesting increased responsiveness to ICIs (Figures 2E and S2A–S2C). Complementing these findings, TIDE analysis revealed a strong positive correlation between ICDRS risk levels and TIDE scores, implying reduced immune evasion capacity in low-risk patients (Figure 2F). This pro-inflammatory immune milieu was further corroborated by predictive anti-PD-L1 response rates derived from the TIDE algorithm, where low-risk patients demonstrated a 1.4-fold higher likelihood of anti-PD-L1 responsiveness compared to their high-risk counterparts (*p* = 0.005; Figure 2G). Beyond prognostic stratification, our multi-platform analysis revealed that the ICDRS model can predict distinct immune microenvironment states and therapeutic susceptibility, which further underscores its potential for guiding precision immuno-oncology strategies.

Based on the selection criteria outlined in the Methods, intersection analysis identified three final candidates: sunitinib, quercetin, and erythromycin (Figure 2H and Table S2). Sunitinib emerged as the most promising candidate due to compelling evidence that it activates antitumor immunity and prevents immune tolerance, making it a rational candidate for combination with immunotherapy.^18^^,^^20^^,^^21^^,^^22^ To investigate interaction characteristics between sunitinib and six ICDRGs-encoded proteins, we performed molecular docking predictions. The results revealed that sunitinib had the highest affinity binding (ΔG = −7.85 kcal/mol) with eIF2α at residues CYS-132, SER-134, and LEU-175 (Figure 2I). Significantly, SER-134 resides in immediate proximity to the phosphorylation site SER-51, suggesting potential allosteric influence on phospho-site conformation. DARTS assays confirmed direct interaction, demonstrating sunitinib-mediated protection of eIF2α from proteolytic degradation (Figure 2J).

### The candidate drug sunitinib triggers ICD in HCC cells

**Figure 3:**
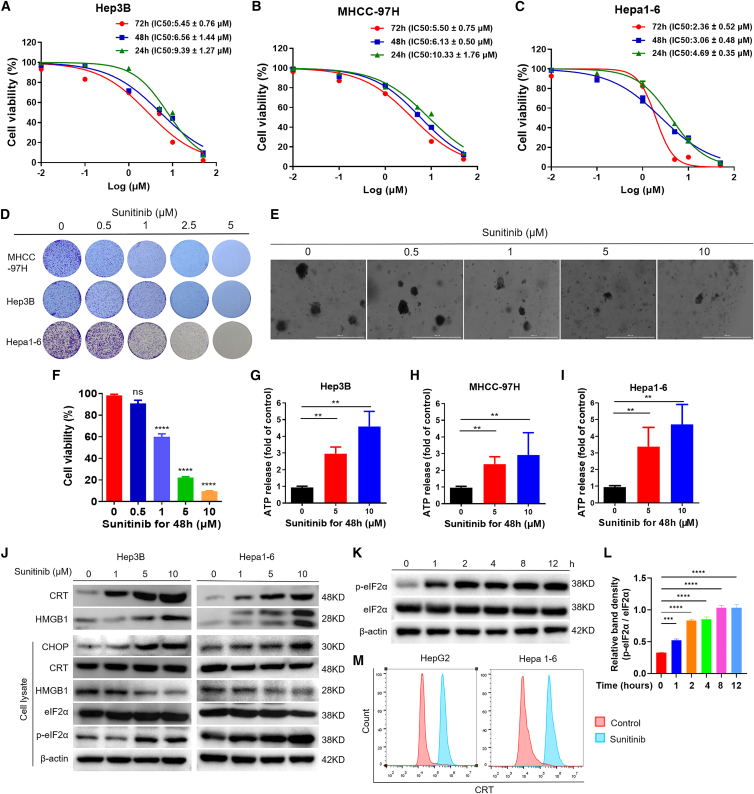
Sunitinib suppresses HCC cell/organoid proliferation and induces ICD-associated DAMP release *in vitro* (A–C) A subsequent analysis of cell viability was conducted using CCK-8 following a time-dependent manner. Each panel displays the HCC cell lines (Hep3B, MHCC-97H, and Hepa 1–6) that were subjected to sunitinib treatment for 24 h, 48 h, and 72 h (*n* = 3). (D) A clone formation assay was performed on HCC cell lines (MHCC-97H, Hep3B, and Hepa1-6) that were treated with different concentrations of sunitinib (0, 0.5, 1, 2.5, and 5 μM). (*n* = 3). (E and F) The HCC organoid growth assay was treated with different concentrations of sunitinib (0, 0.5, 1, 5, and 10 μM). Scale bars: 1000 μm. The quantification of the results is shown in (F). (*n* = 3). (G–I) ATP assay of HCC cell lines (MHCC-97H, Hep3B, and Hepa1-6) was treated with sunitinib at the concentrations and times shown in the figure. (*n* = 3). (J) Signature proteins of ICD in two HCC cell lines were analyzed by Western blot assays. Cells were treated with sunitinib for 24 h for the analysis of membrane CRT and other proteins, while HMGB1 secretion was assessed in the supernatant collected after 48 h of treatment. The unlabeled CRT and HMGB1 signals originate from the cell membrane and the supernatant, respectively. (*n* = 3). (K and L) Immunoblots and its quantitation of phosphorylated eIF2α (*p*-eIF2α) and total eIF2α in Hepa1-6 cells treated by sunitinib for indicated time points. (*n* = 3). (M) Detection of cell surface calreticulin (CRT) exposure by flow cytometry in HCC cells following 24-h sunitinib treatment. (*n* = 3). Data are presented as means ± SD. Statistical analysis was performed using a Student’s *t* test (F–I and L). ∗, *p* < 0.05; ∗∗, *p* < 0.01; ∗∗∗, *p* < 0.001; ∗∗∗∗, *p* < 0.0001.

To explore its anti-tumor effect on HCC, CCK-8 assay and clone formation assay were employed. Results demonstrated time-dependent inhibitory effects of sunitinib on HCC cell proliferation across multiple treatment durations, with progressively decreasing IC50 values (μM) quantified and presented in the corresponding figures (Figures 3A–3C). Clone formation assay confirmed the limitation effect of sunitinib on HCC cell growth in a relatively low-dose (Figure 3D). To further corroborate the anti-HCC efficacy of sunitinib, we conducted validation using patient-derived HCC organoids. The results showed that sunitinib treatment significantly attenuated HCC organoid growth, manifesting as both a marked reduction in 3D tumor spheroid formation capacity and a concomitant decrease in cellular viability (Figures 3E and 3F). Next, we investigated whether sunitinib could induce ICD in HCC. Sunitinib treatment significantly increased the ATP release in supernatant of HCC cell lines in a dose-dependent manner, including Hep3B, MHCC-97H, and Hepa1-6 (Figures 3G–3I). Having established this consistent effect (Figure S3), we next focused on Hepa1-6 and Hep3B cells to delineate the key ICD markers. Accordingly, sunitinib upregulated the protein expression levels of the endoplasmic reticulum stress marker CHOP, HMGB1, and CRT (CRT on the membrane and HMGB1 in the supernatant) in a dose-dependent manner (Figure 3J). In addition, sunitinib treatment significantly induced phosphorylation of eIF2α in Hepa1-6 and Hep3B cells (Figure 3J). This phosphorylation became evident as early as 1 h after treatment and increased steadily over 4 h (Figures 3K and 3L). Flow cytometry confirmed the increased exposure of CRT on the surface of tumor cells after 24 h of sunitinib treatment (Figure 3M), indicating that sunitinib treatment significantly enhanced the immunogenicity of these cells. Overall, our data demonstrate that sunitinib inhibits the viability and proliferation of HCC cells in cell lines and organoids. More importantly, sunitinib treatment induced ICD of tumor cells in a dose-dependent manner as evidenced by the pathognomonic hallmark of eIF2α phosphorylation.

### Sunitinib induces ICD by facilitating eIF2α phosphorylation

**Figure 4:**
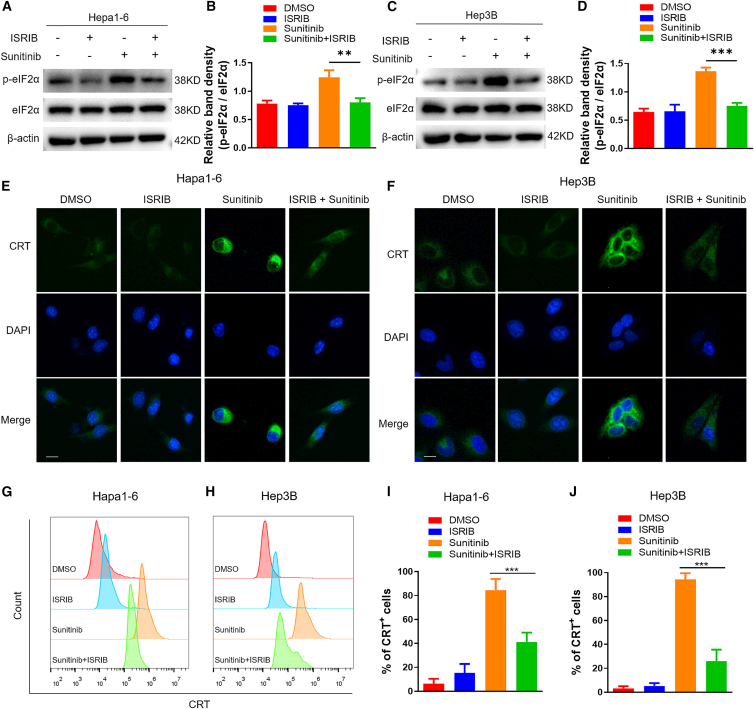
Sunitinib induces ICD in HCC cell lines by facilitating eIF2α phosphorylation (A–D) Hepa 1–6 and Hep3B cells were treated with sunitinib (5 μM) for 6 h, with or without co-treatment with ISRIB (1 μM; added 2 h prior to sunitinib). Protein lysates were analyzed by immunoblot for the indicated proteins, with quantification shown in (B) and (D). (*n* = 3). (E and F) Immunofluorescence cell-surface staining of CRT (green) in Hepa 1–6 and Hep3B treated for 24 h with sunitinib alone or in combination with 1 μM ISRIB. (*n* = 3). Scale bars: 10 μm. (G–J) Flow cytometry was performed to analyze the surface expression of CRT in HCC cells treated with sunitinib for 24 h (*n* = 3). Data are presented as means ± SD. Statistical analysis was performed using a Student’s *t* test (B, D, I, and J). ∗, *p* < 0.05; ∗∗, *p* < 0.01; ∗∗∗, *p* < 0.001.

Previous studies have established that eIF2α phosphorylation is indispensable for the extracellular exposure of CRT, a key DAMP in immunogenic cell death.^23^^,^^24^ Based on our molecular docking results that revealed a direct interaction between sunitinib and eIF2α (Figure 2I), we hypothesized that sunitinib induces ICD by facilitating eIF2α phosphorylation, thereby providing a mechanistic link from target engagement to immunogenic outcomes. To test this, we employed ISRIB, a specific inhibitor that blocks the integrated stress response downstream of *p*-eIF2α. Western blot analysis confirmed that sunitinib-induced eIF2α phosphorylation was significantly attenuated upon ISRIB pretreatment (Figures 4A–4D). Consistent with this, both immunofluorescence and flow cytometry quantification demonstrated that ISRIB pretreatment markedly abrogated sunitinib-induced CRT exposure on the cell surface (Figures 4E–4J). Critically, ISRIB alone at the concentration used did not affect cell viability (Figure S4), ensuring that its effects were not due to cytotoxicity. Taken together, these findings indicate that sunitinib triggers ICD primarily through the activation of the eIF2α phosphorylation pathway.

### Sunitinib enhances dendritic cell maturation and CD8^+^ T cell activation via eIF2α phosphorylation *in vitro*

**Figure 5:**
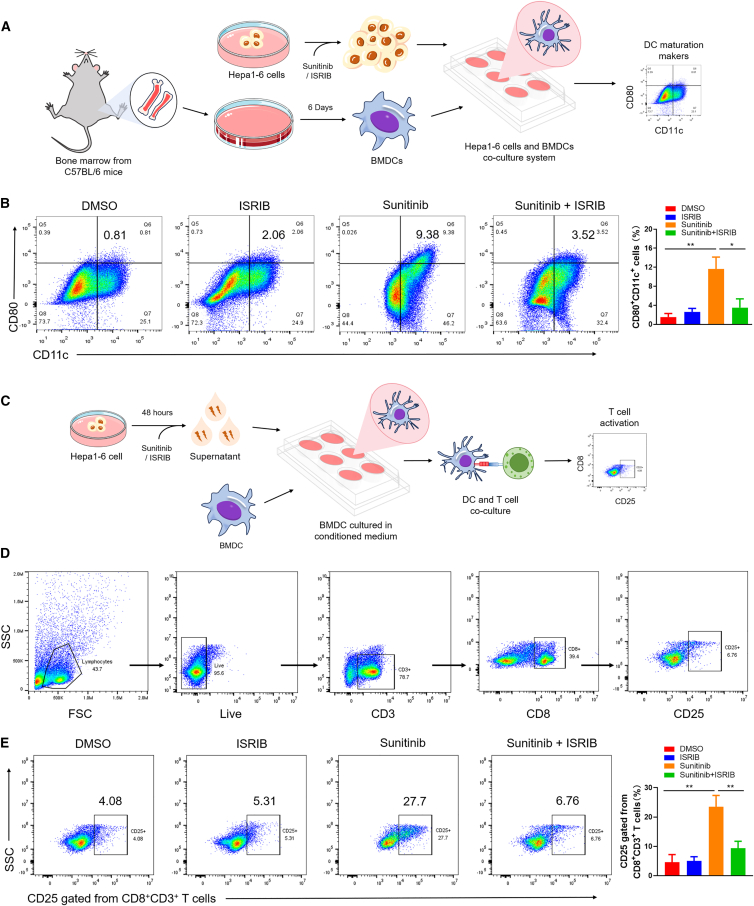
Sunitinib promotes in *ex vivo* DC maturation and CD8^+^ T cell activation by driving phosphorylation of eIF2α (A) Schematic representation of the process for murine DC isolation, differentiation, and maturation analysis. (B) After the sunitinib or/and ISRIB treatment, the treated Hepa 1–6 cells were then co-cultured with immature DC for another 24 h. The CD80 and CD11c positive cells were analyzed by the flow cytometry. (*n* = 3). (C) Schematic representation of the experimental workflow for T cell activation. (D) Gating strategy for Flow cytometry analysis of T cell activation. (E) Analysis of DC-mediated T cell activation *in vitro.* (*n* = 3). Data are presented as means ± SD. Statistical analysis was performed using a Student’s *t* test (B and E). ∗, *p* < 0.05; ∗∗, *p* < 0.01.

Dendritic cells (DCs) are essential for initiating antigen-specific anti-tumor immunity.^25^^,^^26^ To assess whether sunitinib-induced ICD activates DCs, we co-cultured mouse bone marrow-derived DCs (BMDCs) with sunitinib-pretreated Hepa1-6 cells. The scheme was exhibited in the Figure 5A. Flow cytometry analysis revealed that BMDCs exposed to sunitinib-treated cells showed a significant increase in surface CD80 expression on CD11c^+^ cells. This effect was abolished by co-treatment with the integrated stress response inhibitor ISRIB (Figure 5B), indicating dependence on eIF2α phosphorylation. We next asked whether the soluble factors released during ICD could prime CD8^+^ T cell activation. Following the illustrated experimental workflow (Figure 5C), BMDCs were stimulated with conditioned medium from sunitinib-treated Hepa1-6 cells and then co-cultured with CD3^+^ T cells. Accordingly, sunitinib-conditioned medium significantly increased the proportion of activated CD8^+^ T cells (CD3^+^CD8^+^CD25^+^). This enhancement was again attenuated by ISRIB (Figures 5D and 5E). Together, these data demonstrate that sunitinib promotes DC maturation and subsequent CD8^+^ T cell activation through an eIF2α-dependent pathway.

### Sunitinib improves immune protection and memory by inducing ICD via the eIF2α/CRT axis *in vivo*

**Figure 6:**
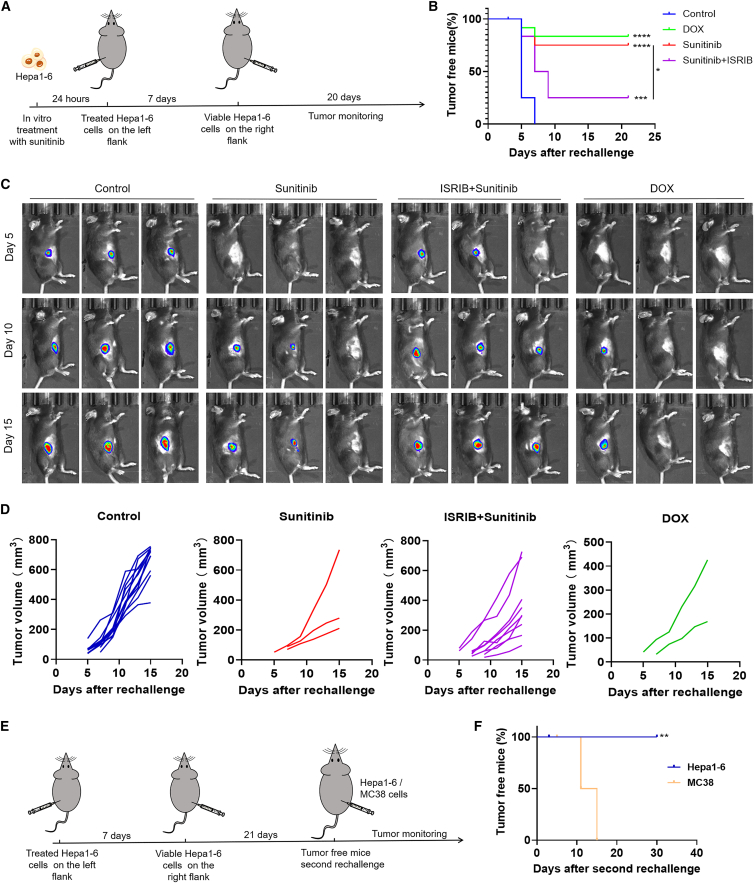
Sunitinib triggers ICD via the eIF2α/CRT Axis *in vivo* (A) Schematic illustration of the vaccination experiment to evaluate the tumor vaccine potential of sunitinib. (B) The tumor free curve of Hepa 1–6 tumor on the right side in mice after the different treatments. (*n* = 12). (C) *In vivo* imaging of tumor growth following viable Hepa1-6 challenge. (D) Individual tumor growth curves of tumor-bearing mice on the right flank of each group. (*n* = 12). (E) Tumor-free mice after receiving sunitinib-treated Hepa1-6 cells received a second rechallenge by subcutaneous injection of growing Hepa1-6 cells (*n* = 5) or MC38 cells (*n* = 4). (F) Percentage of the tumor-free mice after a second tumor rechallenge. The curves for tumor-free mice were generated using the Kaplan-Meier method, and significance between groups was calculated by the log rank test (B and F). ∗, *p* < 0.05; ∗∗, *p* < 0.01; ∗∗∗, *p* < 0.001; ∗∗∗∗, *p* < 0.0001.

On the basis of the gold-standard vaccination assay in immunocompetent, syngeneic mice for evaluating adaptive immunity initiation,^6^ we assessed the *in vivo* ICD activity of sunitinib. Immunocompetent mice were vaccinated with Hepa1-6 cells pretreated with sunitinib, doxorubicin (DOX), ISRIB (an eIF2α phosphorylation inhibitor), ISRIB + sunitinib, or freeze-thaw cycles, followed by a contralateral challenge with untreated Hepa1-6 cells on day 7 (Figure 6A). Sunitinib and DOX groups exhibited robust immune protection (sunitinib: 9/12 tumor-free; DOX: 10/12 tumor-free), whereas freeze-thawed control cells failed to confer protection (0/12 tumor-free) (Figures 6B–6D). Mice vaccinated with sunitinib- or DOX-pretreated cells showed markedly reduced tumor growth, with only a few small tumors developing in a minority of animals. In stark contrast, large tumors formed in all mice of the control group and in the majority of mice co-treated with ISRIB, which effectively reversed the protective effect of sunitinib. Mechanistically, pretreatment with ISRIB reversed the immune protection conferred by sunitinib *in vivo*: the tumor incidence in the ISRIB + sunitinib group (9/12) was significantly higher than that in the sunitinib monotherapy group (3/12, *p* < 0.05), suggesting the eIF2α/CRT axis as a critical mediator of sunitinib-triggered ICD. To further evaluate the immune memory function, the nine tumor-free mice surviving from the sunitinib-treated Hepa1-6 cells were separated into two groups and then received a second rechallenge with Hepa1-6 cells and MC38 cells, respectively, on day 22 (Figure 6E). As shown in Figure 6F, tumor-free mice from the sunitinib group displayed antigen-specific immune memory against homologous Hepa1-6 rechallenge but not heterologous MC38 tumors, underscoring the durability and specificity of the immune response.

### Sunitinib in combination with anti-PD-L1 elicits synergistic anti-HCC efficacy

**Figure 7:**
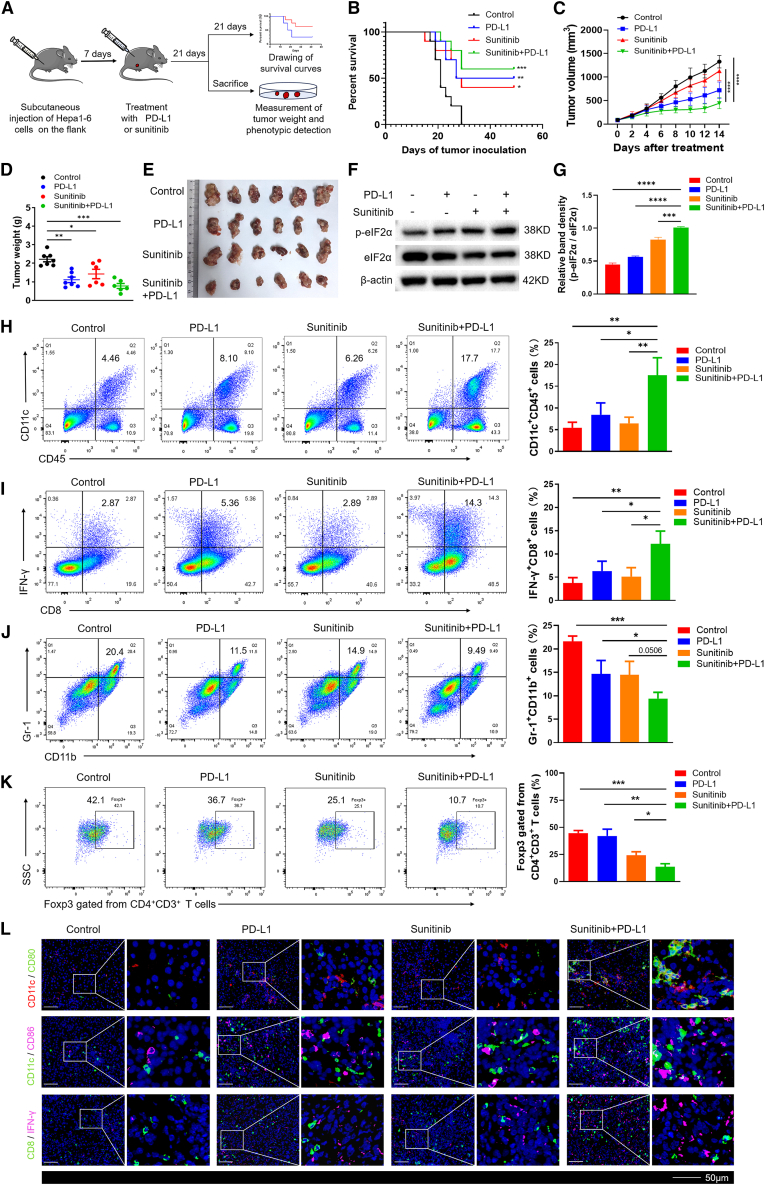
Sunitinib in combination with anti-PD-L1 elicits synergistic anti-HCC efficacy (A) The scheme of tumor incubation and treatment approach. (B and C) Survival curves (B) and tumor growth (C) of Hepa1-6-induced subcutaneous HCC in C57BL/6 mice treated with vehicle or single agent or combination-treated Hepa1-6 cells. (*n* = 8–10). (D) Mice were sacrificed at day 21 after treatment, tumor weight was measured. (E) Representative photographs of dissected tumors from each group. (F) Western blot analysis of *p*-eIF2α and eIF2α protein expression levels in tumors from C. (*n* = 3). (G) Relative band density graphs for *p*-eIF2α normalized to eIF2α from F. (H–K) Flow cytometry plots of percentages of tumor-infiltrating CD45^+^CD11c^+^ DCs (H), CD8^+^IFN-γ^+^ T cells (I), CD11b^+^Gr-1^+^MDSCs (J), and Foxp3^+^CD4^+^CD3^+^ T cells (K) within the tumors following the indicated treatment groups. (*n* = 3). (L) Multiplex immunofluorescence staining of the markers shown in the diagram in Hepa1-6-induced subcutaneous HCC model. (*n* = 3). Scale bars: 50 μm. Survival curves were generated using the Kaplan-Meier method, and statistical significance was assessed by the log rank test (B). Data in C was analyzed by two-way ANOVA. Comparisons between two groups (D and G–K) were performed using an unpaired two-tailed Student’s *t* test. ∗, *p* < 0.05; ∗∗, *p* < 0.01; ∗∗∗, *p* < 0.001; ∗∗∗∗, *p* < 0.0001.

Given the well-established role of ICD inducers in sensitizing tumors to immunotherapy,^7^ we hypothesized that sunitinib would augment the efficacy of anti-PD-L1 therapy. This hypothesis was further supported by our *in vitro* finding that prolonged sunitinib treatment (72 h) significantly downregulated PD-L1 expression on HCC cells (Figure S5), suggesting a potential to alleviate this immune checkpoint. To test the combination *in vivo*, we established subcutaneous Hepa1-6 HCC models in mice. The tumor-bearing mice were then treated with vehicle control, sunitinib, anti-PD-L1, or a combination of sunitinib and anti-PD-L1 (Figure 7A). The combination group exhibited significant survival benefits (Figure 7B) and markedly suppressed tumor growth compared to monotherapies (Figures 7C–7E). Immunoblot analysis revealed elevated *p*-eIF2α expression in the combination group (Figures 6F and 6G), suggesting enhanced ICD.

We next performed immune profiling of the tumor microenvironment. Flow cytometry and multiplex immunofluorescence showed that combination therapy significantly increased the infiltration of activated dendritic cells (CD11c^+^CD45^+^, CD80^+^CD11c^+^, and CD86^+^CD11c^+^) and IFN-γ^+^CD8^+^ T cells compared to all other groups (Figures 7H, 7I, 7L, and S6). In parallel, we analyzed key immunosuppressive cell populations. The combination treatment significantly reduced tumor infiltration of regulatory T cells (Tregs, Foxp3^+^CD4^+^) and myeloid-derived suppressor cells (MDSCs, Gr-1^+^CD11b^+^) relative to the control group (Figures 7J and 7K). Sunitinib monotherapy also reduced these populations, though to a lesser extent. Together, these findings demonstrate that sunitinib plus anti-PD-L1 not only activates anti-tumor immune effectors in HCC, but also alleviates local immunosuppression. This dual action results in a synergistically remodeled and more anti-tumorigenic immune microenvironment.

## Discussion

Hepatocellular carcinoma (HCC) remains a formidable clinical challenge due to its immunosuppressive tumor microenvironment (TME) and resistance to conventional therapies.^3^^,^^27^ Our study demonstrates that immunogenic cell death (ICD) induction, mediated through the eIF2α phosphorylation (*p*-eIF2α) axis, holds promise for overcoming these barriers. By integrating multi-omics analyses, pharmacological screening, and functional validation, we identified sunitinib-a multi-targeted tyrosine kinase inhibitor (TKI)-as a novel ICD inducer capable of remodeling the HCC TME and synergizing with PD-L1 blockade. These findings reveal a non-canonical immunomodulatory axis of sunitinib that is mediated through eIF2α-dependent stress response signaling. This work thereby establishes a clinically actionable blueprint for repurposing selective kinase inhibitors as ICD potentiators. Such repurposing establishes a new paradigm for combination therapy with PD-L1 blockade in hepatocellular carcinoma.

HCC sustains its immunosuppressive niche through defective dendritic cell activation, clonally exhausted T cell populations, and maladaptive stress response signaling,^2^^,^^3^^,^^4^ collectively establishing a permissive microenvironment for immune evasion. Our six-gene ICDRS, derived from transcriptomic profiling of TCGA-LIHC and validated in external cohorts, effectively stratifies HCC patients into prognostically distinct subgroups. Low-risk patients exhibited enhanced immune infiltration, elevated antigen-presenting cell activity, and reduced immune evasion markers, aligning with the concept that ICD primes the TME for anti-tumor immunity. Importantly, the ICDRS model demonstrated predictive accuracy for immune checkpoint inhibitor (ICI) responsiveness, as evidenced by higher IPS and lower tumor immune dysfunction and exclusion (TIDE) scores in low-risk subgroups. This immunologically active profile correlated with a “hot” TME, characterized by enriched infiltration of cytotoxic CD8^+^ T lymphocytes, gamma-delta T cells, and activated DCs. These are all hallmarks of effective anti-tumor immunity. We also observed significantly lower TMB in the low-risk group compared to the high-risk group, suggesting that higher TMB was not associated with increased immune cell infiltration, a finding consistent with previous reports in HCC that substantial copy number alterations lead to impaired antigen presentation mechanisms.^28^^,^^29^ Mechanistically, ICDRS-defined immune activation (e.g., enhanced antigen cross-presentation and T cell recruitment) could restore DC-dependent T cell priming. Furthermore, the dynamic nomogram integrating ICDRS with clinical parameters offers a practical tool for risk stratification and therapeutic decision-making in heterogeneous HCC populations.

Through multi-platform pharmacological screening and molecular docking, we identified sunitinib as a high-affinity binder of eIF2α, the master regulator of the integrated stress response (ISR). We found that sunitinib potently induces eIF2α phosphorylation, initiating a cascade of immunogenic signals. The sustained activation of this pathway was responsible for the subsequent exposure of calreticulin (CRT) and release of ATP/HMGB1—key hallmarks of ICD. These effects were dose-dependent: low concentrations elicited immunogenic stress, while high concentrations may cause passive DAMP release due to increased membrane permeabilization. Sunitinib-induced ICD follows a defined temporal sequence. Rapid and sustained eIF2α phosphorylation triggers the integrated stress response. This leads to CRT translocation to the cell surface within 24 h. Subsequently, the release of soluble “find-me” signals like ATP and HMGB1 peaks around 48 h. This ordered event ensures effective DC recruitment and activation, thereby bridging innate and adaptive immunity. Crucially, the eIF2α inhibitor ISRIB markedly reduced sunitinib-induced CRT translocation, confirming that *p*-eIF2α-mediated signaling is essential for sunitinib-induced ICD. This finding aligns with prior studies linking sustained eIF2α phosphorylation to enhanced antigen presentation and T cell activation.^13^^,^^30^ Notably, this dual role of kinase inhibition and ICD induction is not unique to sunitinib; certain TKIs, including crizotinib and cabozantinib, can also act as bona fide inducers,^31^^,^^32^ suggesting that some TKIs may constitute a promising class of ICD-inducing agents. Beyond its role as a multi-kinase inhibitor, sunitinib triggers ICD through a mechanism shared with other agents, namely, the induction of ER stress. As prior studies have emphasized, ROS-mediated ER stress plays a key role in inducing ICD by various agents in HCC models.^33^^,^^34^^,^^35^^,^^36^ Similarly, our data show that sunitinib elicited ER stress, as marked by increased CHOP expression (Figure 3J). Furthermore, induction therapy with other ICD-inducing chemotherapeutics, such as doxorubicin and oxaliplatin, has been shown to sensitize malignant lesions to ICIs.^7^^,^^37^ These collective findings underscore the broader potential of combining ICD inducers (including selective TKIs) with immunotherapeutic strategies.

The translational relevance of these mechanisms was further validated *in vivo*. Vaccination with sunitinib-pretreated HCC cells conferred durable, antigen-specific immune protection in immunocompetent mice, a phenomenon partially reversed by ISRIB co-treatment. This residual protection suggests additional sunitinib-mediated immunomodulatory pathways beyond the eIF2α/CRT axis, such as the downregulation of PD-L1 on HCC cells we observed *in vitro* (Figure S5). To mitigate the documented toxicities of sunitinib, a dosage equivalent to half of the clinical dose was employed in our study, a strategy supported by previous literature.^18^^,^^38^ Notably, combining sunitinib with PD-L1 blockade achieved synergistic tumor regression in subcutaneous HCC models, primarily via ICD initiation, with the aforementioned PD-L1 reduction providing a complementary mechanism. This combination therapy expanded mature DC populations and reinvigorated IFNγ^+^CD8^+^ T cell infiltration. In parallel, the combination regimen also reduced the accumulation of Tregs and MDSCs within the TME, effectively counteracting two major resistance pathways to immunotherapy. These findings directly demonstrate that sunitinib-triggered ICD can remodel the immunosuppressive HCC microenvironment and potentiate anti-PD-L1 therapy.^37^^,^^39^ Critically, sunitinib may offer a more precise therapeutic approach compared to classical ICD inducers. Its defined action through eIF2α phosphorylation contrasts with the broader mechanisms and associated toxicities of agents like doxorubicin and oxaliplatin. This aligns with the development of other novel ICD-inducing strategies, including new pharmacological agents like G4P-C7A^39^ and innovative bio-synthetic platforms such as engineered nanovaccines,^40^^,^^41^^,^^42^ all aiming to more precisely remodel the tumor microenvironment for immunotherapy. Our findings extend these paradigms and position sunitinib as an immune-potentiating agent for combinatorial regimens. To elucidate the underlying rewiring, future single-cell studies could provide deeper insights into the tumor immune ecosystem elicited by this combination therapy.

In conclusion, our study repositions sunitinib as a bona fide inducer of ICD in HCC via the eIF2α phosphorylation axis. By activating this pathway, sunitinib enhances dendritic cell priming and IFN-γ^+^ CD8^+^ T cells infiltration, thereby fostering an immunostimulatory tumor microenvironment. This immunomodulatory action makes sunitinib a particularly suitable partner for combination with ICIs. Crucially, we integrate this drug-repurposing strategy with an ICDRS to propose a dual-component clinical solution: the ICDRS biomarker stratifies patients likely to benefit, while sunitinib-triggered ICD converts immunologically “cold” tumors into ICI-responsive ones. This paradigm not only offers a cost-effective therapeutic avenue but also establishes a framework for precision immunotherapy in HCC and beyond.

### Limitations of the study

Although our study shows that sunitinib-induced immunogenic cell death (ICD) synergized with anti-PD-L1 therapy in murine HCC models, two translational challenges merit attention. First, the long-term safety and efficacy of “sunitinib + anti-PD-L1” combination therapy in humans remains to be determined due to the immunosuppressive landscape of human hepatocellular carcinoma being more complex. Secondly, the application of our ICD-related risk signature (ICDRS) requires validation in prospective trials and standardization to overcome clinical challenges such as sample source variability and batch effects in multi-center settings. Third, our mechanistic and functional studies on immune activation relied on murine dendritic and T cells, and further validation in human immune cells would strengthen the clinical relevance of our findings.

## Resource availability

### Lead contact

Further information and requests for resources and reagents should be directed to and will be fulfilled by the lead contact, Ting Liu (liuting@gzucm.edu.cn).

### Materials availability

This study did not generate new unique reagents.

### Data and code availability

• The multi-omics data used in this study were obtained from public repositories. Transcriptomic and genomic data for the TCGA-LIHC cohort (n = 371), including RNA-seq, somatic mutations, and copy number alterations, were downloaded from the TCGA data portal (https://portal.gdc.cancer.gov/). Gene expression data from the GEO-GSE14520 dataset (Affymetrix U133A array, n = 223) were acquired from the GEO database (https://www.ncbi.nlm.nih.gov/geo/). Additionally, RNA-seq data from the ICGC-LIRI-JP cohort (n = 234) were retrieved from the ICGC database (https://dcc.icgc.org/projects/LIRI-JP).
• This paper does not report original code.
• Any additional information required to reanalyze the data reported in this work paper is available from the lead contact upon request.

## Acknowledgments

The National Natural Science Foundation of China (nos. 82574817, and 82202986), The Natural Science Foundation of Guangdong Province (nos. 2024A1515012867, 2023A1515220017, and 2025A1515010135), Young Doctor “Sailing” Project of Science and Technology Department of Guangzhou (no. 2024A04J3291), Outstanding Young Talents Seedling Program of Guangdong Academy of Traditional Chinese Medicine (no. SZ2023QN03), Scientific Research Project of Guangzhou Traditional Chinese Medicine Bureau (nos. 20252033 and 20242034) and National Nature Cultivation Project of Guangdong Hospital of Traditional Chinese Medicine (no. YN2024GZRPY061), 10.13039/501100010256Guangzhou Municipal Science and Technology Project (no. 2025A03J0466).

## Author contributions

Writing – original draft, methodology, conceptualization, software, data curation: Y.G.; writing – original draft, software, methodology, data curation: H.M.; software, formal analysis, validation: P.H.; software, data curation: H.Y.; formal analysis, data curation: M.S.; software, data curation: H.G.; software, data curation, validation: C.S.; writing – review and editing, validation: Y.O.; writing – review and editing, resources, supervision, funding acquisition: J.T.; writing – review and editing, resources, supervision: X.H.; conceptualization, writing – review and editing, writing – original draft, resources, validation, project administration, funding acquisition: T.L.

## Declaration of interests

The authors declare no competing interests.

## STAR★Methods

### Key resources table

**Table:** 

REAGENT or RESOURCE	SOURCE	IDENTIFIER
**Antibodies**		

eIF2α	Selleck Chemicals	Cat#F0316
Phospho-eIF2α (Ser51)	Selleck Chemicals	Cat#F0257
PERK	Selleck Chemicals	Cat# F0027
Phospho-PERK (Thr980)	Cell Signaling Technology	Cat#3179; RRID:AB_2095853
Calreticulin	ImmunoWay	Cat#YT0620
HMGB1	ImmunoWay	Cat#YM8327
β-actin	ImmunoWay	Cat#YM8343
PD-L1/CD274	Proteintech	Cat#28076-1-AP; RRID:AB_2881052
CHOP/GADD153	Proteintech	Cat#15204-1-AP; RRID:AB_2292610
PE anti-mouse CD11c	BioLegend	Cat#117307; RRID:AB_313776
Pacific Blue anti-mouse CD80	BioLegend	Cat#104723; RRID:AB_2076001
PerCP/Cyanine5.5 anti-mouse CD3	BioLegend	Cat#100218; RRID:AB_1595492
FITC anti-mouse CD8a	BioLegend	Cat# 100803; RRID:AB_312764
PE anti-mouse CD45	BioLegend	Cat#147711; RRID:AB_2563597
PE anti-mouse IFN-γ	BioLegend	Cat#505808; RRID:AB_315402
PerCP/Cyanine5.5 anti-mouse Ly-6G/Ly-6C (Gr-1)	BioLegend	Cat#108428; RRID:AB_893558
PE/Cyanine7 anti-mouse CD25	BioLegend	Cat#102015; RRID:AB_312864
PE anti-mouse FOXP3	BioLegend	Cat#126404; RRID:AB_1089117
anti-mouse CD16/32	Biolegend	Cat#101319; RRID:AB_1574973
Rabbit anti-mouse CD8 alpha	Abcam	Cat#ab209775
Alexa Fluor® 488 Anti-Calreticulin	Abcam	Cat#ab196158
Rabbit anti-mouse IFN-γ antibody	Affinity	Cat#DF6045
Rabbit anti-mouse CD11c antibody	Affinity	Cat#DF7585
Rabbit anti-mouse CD86 antibody	Affinity	Cat#DF6332
Rabbit anti-mouse CD80 antibody	Affinity	Cat#DF7682

**Chemicals, peptides, and recombinant proteins**		

Sunitinib (SU11248)	Selleck Chemicals	Cat#S7781
anti-PD-L1	Selleck Chemicals	Cat#A2115
ISRIB (Integrated Stress Response Inhibitor)	Selleck Chemicals	Cat#S0706
Doxorubicin	Selleck Chemicals	Cat#E2516
Oxaliplatin	Selleck Chemicals	Cat#S1224
D-luciferin	AbMole	Cat# M8873
RPMI-1640 Medium	Thermo Fisher Scientific-Gibco	Cat#C11995500BT
Dulbecco’s Modified Eagle Medium	Thermo Fisher Scientific-Gibco	Cat#11054001
penicillin/streptomycin	Thermo Fisher Scientific-Gibco	Cat#15140122
Proteinase K	Sigma-Aldrich	Cat#P2308
GM-CSF	PeproTech	Cat#315-03-20UG
IL-4	PeproTech	Cat#214-14-5UG

**Critical commercial assays**		

Cell Counting Kit-8 (CCK-8)	GlpBio	Cat#GK10001
ATP Assay Kit	Beyotime Biotechnology	Cat#S0027
BCA protein assay kit	Fdbio science	Cat#FD2001
Zombie NIR Fixable Viability Kit	Biolegend	Cat#423105
Mouse CD3^+^ T cell Isolation Kit	Selleck Chemicals	Cat#B90021
Lipofectamine3000	Thermo Fisher Scientific-Invitrogen	Cat#L3000008

**Deposited data**		

RNA-seq data	TCGA-LIHC	https://portal.gdc.cancer.gov/
RNA-seq data	ICGC-LIRI-JP	https://dcc.icgc.org/projects/LIRI-JP
RNA-seq data	GEO	GSE14520
Drug-Gene Interaction Data	CMap	https://clue.io/
Drug-Gene Interaction Data	L1000 FWD	https://maayanlab.cloud/l1000fwd/
Drug-Gene Interaction Data	DGIdb	https://dgidb.org/

**Experimental models: Cell lines**		

Hepa1-6	ATCC	RRID: CVCL_0327
MHCC-97H	ATCC	RRID: CVCL_4972
Hep3B	ATCC	RRID: CVCL_0326
MC38	Cell Bank of the Chinese Academy of Sciences	RRID: CVCL_B288

**Experimental models: Organisms/strains**		

C57BL/6J male mice	Guangdong Medical Laboratory Animal Center	RRID: IMSR_JAX:000664

**Software and algorithms**		

GraphPad Prism 9.5	GraphPad software	https://www.graphpad.com
ImageJ 1.54g	ImageJ software	https://imagej.nih.gov/
R software 4.4.2	R Core Team	https://www.r-project.org/
PyMOL 2.6	Schrödinger	https://pymol.org/
Imaris 9.0.1	Oxford Instruments	https://imaris.oxinst.com/

### Experimental model and study participant details

#### Data collection and multi-omics integration

The ICD-related gene set (112 genes, Table S3) was curated from literature.^14^^,^^43^ Multi-omics integration included: (1) TCGA-LIHC cohort (*n* = 371 samples) with FPKM -normalized RNA-seq, somatic mutations, and copy number alterations; (2) GEO-GSE14520 expression data (Affymetrix U133A array, *n* = 223 samples); (3) ICGC-LIRI-JP dataset (*n* = 234 samples) with FPKM -normalized RNA-seq. All datasets are publicly available: TCGA-LIHC (https://portal.gdc.cancer.gov/), GEO-GSE14520 (https://www.ncbi.nlm.nih.gov/geo/), and ICGC-LIRI-JP (https://dcc.icgc.org/projects/LIRI-JP).

#### HCC cell line culture and organoid maintenance

HCC cell lines (Hepa1-6, Hep3B, and MHCC-97H) were obtained from ATCC and cultured in Dulbecco’s modified Eagle’s medium (DMEM; Cat#11054001, Gibco) supplemented with 10% fetal bovine serum (FBS; Cat#FS201-02, TransGen) and 100 U/ml penicillin/streptomycin (Cat#15140122, Gibco) at 37°C under 5% CO_2_.

For HCC organoids, cryopreservation, thawing, and culture protocols were adapted from Liu et al.^44^ Briefly, cryopreserved organoids (not exceed five generations before using) stored in liquid nitrogen were rapidly thawed in a 37°C water bath for 3 min. The thawed organoid suspension was mixed with an equal volume of growth factor-reduced Matrigel (Cat#354230, Corning) on ice, and droplets were plated into 24-well plates. Following polymerization at 37°C, culture medium was added to each well. Organoids were maintained in a humidified incubator (37°C, 5% CO_2_) with medium replacement every 48 h. Morphological recovery was assessed daily using bright-field microscopy; only organoids with intact luminal structures and diameters >50 μm after 5 days of recovery were utilized for subsequent experiments. For human-derived HCC organoids used in this study, the original patient biopsies were collected at The First Affiliated Hospital of Sun Yat-sen University between 2017 and 2021 with informed consent and under ethical approval (No. 2018 [43]).

#### Mouse experiment

Male C57BL/6J mice (6–8 weeks) were obtained from Guangdong Medical Laboratory Animal Center (Guangzhou, China). The vaccination assays were performed using combination-treated Hepa1-6 cells. Briefly, 1 × 10^6^ Hepa1-6 cells were treated with 5 μM sunitinib, 1 μM ISRIB, or 1.6 μM doxorubicin for 24 h, while control cells received equivalent volumes of drug solvent. After treatment, the cells were subcutaneously injected into the left flank of male C57 mice (12 mice per group). Seven days later, mice were challenged with viable (i.e., growing) Hepa1-6 cells with luciferase (6 × 10^5^ cells in 100 μL sterile PBS) on their right flank. Tumor growth was monitored by *in vivo* bioluminescence imaging after D-luciferin (150 mg/kg, Cat# M8873, AbMole) injection on days 5, 10, and 15. The investigator responsible for caliper measurements and calculating tumor volume (using the formula: Volume = (length × width^2^)/2) was kept unaware of the group allocation and treatment regimen for each mouse. Tumor-free survival curves were concurrently recorded. Experiments were stopped once the tumor volume >2000 mm^3^ or >20% body weight loss.

To assess the ability of sunitinib to enhance tumor sensitivity to immune checkpoint blockade, primary tumors were established by subcutaneous injection of Hepa1-6 cells (1×10^6^ cells/mouse) into the flank region of male C57BL/6J mice. When tumor volumes reached approximately 50 mm^3^, tumor-bearing mice were randomly assigned to the control or treatment groups (*n* = 20) using a computer-generated randomization scheme created with GraphPad Prism software (version 9.5). The cohorts were as follows: sunitinib monotherapy (20 mg/kg orally, 5 days/week, Cat#S7781, Selleck), anti-PD-L1 monotherapy (10 mg/kg intraperitoneally, 3 days/week, Cat#A2115, Selleck), sunitinib + anti-PD-L1 combination therapy (sunitinib administered 3 days prior to PD-L1), and vehicle control (0.5% carboxymethylcellulose orally + PBS intraperitoneally). At treatment initiation (day 0), each group was stratified into two subgroups (n = 8–10 per subgroup) with matched tumor volumes. Both subgroups received identical drug regimens for the full 3-week duration. The survival monitoring subgroup was observed for an additional 21 days post-treatment (total 49 days from day of tumor inoculation) with predefined humane endpoints. The terminal analysis subgroup was euthanized immediately after the 3-week treatment period (day 21) for tumor excision. Excised tumors were divided into three portions: Western blot for *p*-eIF2α expression, multiple immunofluorescence staining, and flow cytometry for immune infiltration analysis. We did not observe any treatment-related adverse events. All animal experiments in this study were conducted in accordance with the guidelines approved by the Welfare and Ethical Committee for Experimental Animal Care of Guangzhou University of Chinese Medicine (Approval No. 20240417001).

#### Ethics approval

All animal experiments in this study were conducted in accordance with the guidelines approved by the Welfare and Ethical Committee for Experimental Animal Care of Guangzhou University of Chinese Medicine (approval no. 20240417001). For human-derived HCC organoids, the original patient biopsies were collected from The First Affiliated Hospital of Sun Yat-sen University between 2017 and 2021 under ethical approval (no. 2018 [43]) with informed consent. The cryopreserved organoids utilized in this study were derived from these clinically approved samples and maintained under the ethical framework of the original protocol.

### Method details

#### Bioinformatic analysis

First, we analyzed the FPKM transcriptome data in the TCGA-LIHC dataset using the R package “limma”, with screening thresholds set at *p* < 0.01 and |fold change| > 1.2 to derive ICD-related DEGs. Subsequently, Gene Ontology (GO) enrichment analysis was conducted to elucidate the biological pathways associated with these DEGs. Prognosis-associated ICDRGs were initially screened through univariate Cox proportional hazards analysis. A two-step regularization approach was implemented: LASSO regression (10-fold cross-validation, optimal λ selected via λ. min criterion) was applied to reduce multicollinearity, followed by multivariate Cox analysis to identify independently prognostic ICDRGs. The finalized ICD-related risk signature (ICDRS) was computed via the “glmnet” R package using the formula: $risk\;score=\sum_{i=1}^{n}Coefi*xi$, where *n* denotes the number of prognostic ICDRGs, *Coefi* the coefficients, and *xi* the corresponding expression values. Using the median risk score, 371 TCGA-LIHC tumors were stratified into high- and low-risk groups, with Kaplan-Meier analysis comparing their survival outcomes. Time-dependent ROC curves quantified the model’s predictive accuracy. For external validation, the GSE14520 and LIRI-JP datasets were analyzed. To assess clinical relevance, univariate and multivariate Cox analyses evaluated the prognostic value of risk scores against clinicopathological features. Furthermore, a nomogram integrating risk scores and clinicopathological parameters was developed using the “rms” R package to predict 1-, 3-, and 5-year survival rates, while calibration curves verified its precision. To characterize the immune status and immunoreactivity of the samples in the ICDRS subgroup, TIDE analysis (http://tide.dfci.harvard.edu/) and quantification of immunophenoscore (IPS) (https://tcia.at/) were performed for TCGA-LIHC samples. Subsequently, “ggplot2” visualized IPS and TIDE distributions between risk groups.

#### Identification of ICD-inducing agents and molecular docking

To identify potential ICD-inducing drugs against HCC, DEGs were analyzed in this study through three separate platforms for drug prediction: Connectivity Map (CMap), L1000 Fireworks Display (L1000 FWD), and Drug-Gene Interaction Database (DGIdb). First, candidate compounds common to all three databases were identified: compounds with significant negative connectivity scores (score ≤ −80) from the CMap analysis indicating their potential to restore immune activation gene expression while antagonizing pro-tumorigenic pathways, candidate drugs from the L1000 FWD screening with similarity scores ≤ −0.05 threshold that potentially regulate ICD-related pathways, and FDA-approved drugs with tumor/immune-modulatory targets from the DGIdb database. Subsequent prioritization among these candidates was based on a review of existing literature, focusing on their documented mechanisms of action and relevance to ICD induction within the immunosuppressive tumor microenvironment (TME) of HCC. Further validation was performed through molecular docking using AutoDock Vina (v1.5.7) to assess binding affinity between sunitinib and the core ICD proteins, with a binding free energy threshold of < −5.0 kcal/mol defining strong interactions. Representative binding conformations were visualized using PyMOL software.

#### Cell and organoid viability assay

Cell viability was assessed using the Cell Counting Kit-8 (CCK-8) (Cat#GK10001, GlpBio). Briefly, cells were seeded into 96-well plates at a density of 5×10^3^ cells/well in 100 μL of complete medium and incubated for 24 h (37°C, 5% CO_2_). Cells were treated with sunitinib or vehicle control for 24 h. After treatment, 10 μL of CCK-8 reagent was added to each well and incubation was continued for 4 h. Absorbance was measured at 450 nm using a microplate reader. The half-maximal inhibitory concentration (IC50) was calculated by fitting the dose-response data to a log(inhibitor) vs. response (variable slope) model. For the colony formation assay, cells were seeded in 6-well plates at 1×10^3^ cells per well and treated with various doses of sunitinib and cultured for 7 days. The cultured cells were fixed with 4% paraformaldehyde in PBS for 15 min and then stained with 0.5% crystal violet for 5 min.

To evaluate the impact of sunitinib on organoid viability, the organoids were treated with escalating concentrations of the drug (0.5 μM, 1 μM, 5 μM, and 10 μM), with DMSO serving as the vehicle control. All groups were cultured for 48 h. After treatment, organoid viability was quantified using the CellTiter-Glo 3D Cell Viability Assay (Cat#G9681, Promega) according to the protocol of the manufacturer. The luminescence signal was detected using the Full Wavelength Enzyme Labeler (BioTek Epoch, Winooski, Vermont, USA).

#### Drug affinity responsive target stability (DARTS) assay

The cell lysates were aliquoted in equivalent volumes containing 50 μg of protein and incubated for 60 min at room temperature with or without sunitinib (50 μM). Proteinase K from Engyodontium album (Cat#P2308, Sigma-Aldrich) was diluted with TNC buffer to form a working solution with a concentration gradient (0.01, 0.1, 0.5, 1, 5, 10 μg/mL). Then, it was added to all samples at different concentrations and incubated at room temperature for 10 min. The samples were then detected via Western blotting.

#### Determination of ATP secretion

Extracellular ATP levels were detected through an Enhanced ATP Assay Kit (Cat#S0027, Beyotime Biotechnology) following the manufacturer’s protocol. The supernatants of hepatocellular carcinoma cells were harvested after treated with increasing concentrations of Sunitinib at indicated time points. Cell debris was removed by centrifugation. And chemiluminescence derived from ATP was detected on a Microplate Reader (Bio-Rad, Hercules, CA, USA).

#### Western blot analysis

Total proteins from cells and tumor tissues were extracted using RIPA lysis buffer (Cat#FD009, Fdbio science) containing protease inhibitor cocktail (Cat#FD1001, Fdbio science) and phosphatase inhibitor cocktail (Cat#FD1002, Fdbio science). The tumor tissues were homogenized by manual grinding with a grinding tube. Protein levels were quantified using the BCA protein assay kit (Cat#FD2001, Fdbio science). Equal amounts of protein in each group were separated by 10% SDS-PAGE and transferred to poly-vi-nylidene difluoride (PVDF) transfer membranes. The blots were blocked with freshly prepared 5% BSA in TBST for 2 h at room temperature and then incubated with specific corresponding diluted antibodies at 4°C overnight. After three washes with TBST, the membranes were incubated with HRP-conjugated secondary antibody for 2 h at room temperature. The protein bands were visualized via ECL (Cat#FD8020, Fdbio science). Primary antibodies were used at a dilution of 1:1000. Details regarding antibody sources and catalog numbers are listed in key resources table.

#### Immunofluorescence assay

Cells (Hep3B and Hepa 1–6) were cultured on coverslips in 24-well culture plates (10,000 cells per well). After attachment, the cells were treated with sunitinib and cultured for 12 h. Pre-treatment with ISRIB (1 μM) for 2h before dosing if necessary. Cells cultured on coverslips were washed with cold PBS and fixed with 4% paraformaldehyde in PBS for 15 min. After cells were washed three times with PBST (PBS +0.1% Tween 20), cells were further treated with 0.2% Triton X-100 in PBS for 10 min at room temperature, and then cells were blocked in blocking buffer (3% BSA in PBST) for 1 h. The cells were then incubated with Alexa Fluor 488 anti-calreticulin antibody [EPR3924] (Cat#ab196158, Abcam) diluted (1:1,000) in PBST overnight at 4 C. The cells were then washed three times for 5 min in PBST and counterstained with 0.01 g/L DAPI (Cat#D1306, Thermo Fisher) for 5 min. Finally, the cells were rinsed in PBS and imaged with a fluorescence microscope (Leica, DMIL LED, Porto, Portugal).

#### Isolation of bone marrow–derived cells

Bone marrow cells were obtained using a previously described method.^45^ Male C57BL/6J mice inhaled 5% isoflurane prior to cervical dislocation. Mice were immersed in 75% ethanol before separating the muscle tissue around the femur and tibia. The ends of the bones then were cut with scissors and the marrow was rinsed with FBS-free RPMI-1640 (Cat#C11995500BT, Gibco) medium in a Petri dish. Primary cells were cultured in the RPMI-1640 medium supplemented with 40 ng/mL recombinant mouse GM-CSF (Cat#315-03-20UG, PeproTech) and 20 ng/mL recombinant mouse IL-4 (Cat#214-14-5UG, PeproTech). On the third day, the culture was half replaced with fresh culture medium. Immature DCs were harvested after seven days of incubation.

#### DC maturation and T cell activation assays

To assess DC maturation, BMDCs were co-cultured directly with Hepa1-6 cells that had been pre-treated for 24 h with sunitinib (5 μM), sunitinib plus ISRIB (1 μM), or vehicle control. After co-culture, BMDCs were harvested and analyzed for surface expression of the maturation marker by flow cytometry. For the T cell activation assays, conditioned medium was collected from Hepa1-6 cells treated for 48 h with sunitinib, sunitinib plus ISRIB, or vehicle. BMDCs were primed with the respective conditioned medium overnight, washed, and then co-cultured with CD3^+^ T cells isolated from spleens of C57BL/6 mice. DCs and T cells were co-cultured at a 1:5 ratio for 5 days, after which T cell activation was assessed using flow cytometry.

#### Tumor single cell suspension preparation

For analysis of immune cell populations *in vivo*, minced tumors were enzymatically digested by a cocktail of 5 mg/mL Dispase II (Cat#42613-33-2, Sigma-Aldrich), 2.5 mg/mL Collagenase D (Cat#11088866001, Sigma-Aldrich), and 2.5 mg/mL Collagenase type II (Cat# 07419, Stemcell) in PBS (Cat#10010023, Gibco). Single-cell suspensions were filtered using 70-μm nylon cell filters. The filtered cell suspension was centrifuged and resuspended in Erythrocyte Lysis Buffer (Cat#00430054, Invitrogen) for 6 min, and lysis was immediately interrupted by the addition of 10 times the volume of erythrocyte lysate in PBS buffer containing 0.5% FBS. Cells were centrifuged and resuspended in PBS buffer for subsequent experiments.

#### Flow cytometry analysis

Flow cytometry was used to measure CRT surface expression on Hepa1-6 cells, quantify mature DCs and activated CD8^+^ T cells in co-cultures, and profile immune cell infiltration in tumor-derived single-cell suspensions. Cells were harvested and resuspended in PBS buffer. Zombie NIR Fixable Viability Kit (Cat#423105, Biolegend) was firstly added for 30 min. Mouse Fc-Receptors were then blocked with anti-mouse CD16/32 (Cat#101319, Biolegend) for 30 min before antibody staining. Following antibodies for cell surface staining were added for 30 min. Following incubation, cells were washed for intracellular staining. Cells were then fixed with Fixation Buffer (Cat#420801, BioLegend) and permeabilized with Permeabilization Buffer (Cat#00-8333-56, Invitrogen). Cells were washed before intracellular staining with the antibody. Cell phenotypes were analyzed using a Gallios flow cytometer (Beckman Coulter, Miami, Florida, USA), and data were analyzed using FlowJo version 10.8.1.

#### Multiple immunofluorescence staining

For immunofluorescence, sections of formalin-fixed, paraffin-embedded mouse tumors were deparaffinized in xylene, rehydrated through an alcohol gradient, and subjected to antigen retrieval in EDTA buffer (pH 8.0). The sections were then incubated with goat serum for 30 min at 37°C, primary antibodies overnight at 4°C, and secondary antibodies for 45 min at 37°C. For details, all primary antibodies were rabbit-derived Mabs, and all secondary antibodies were goat anti-rabbit IgG H&L (HRP) (Cat#ab205718, Abcam). After staining with the primary and secondary antibodies, the corresponding TSA reagents were used for color development. The primary antibodies and corresponding TSA reagents are available at Table S4. DAPI (Cat# D1306, Thermo Fisher) was used for nuclear staining. Fluorescence signals were detected by laser scanning confocal microscopy (ZEISS LSM 800, Jena, Germany). To quantify the immunofluorescence results, the images were further analyzed using Imaris software (version 7.4, BITPLANE). The “Spot” function was used to locate and enumerate cells based on size and intensity threshold. Alternatively, the absolute number of cells spotted per mm2 in nine high-power fields of interest was statistically analyzed (three separate fields from each mouse and three mice from each group).

### Quantification and statistical analysis

Data in this study are presented as mean ± standard deviation (SD). Relative grayscale intensities in the Western blot bands were quantified via ImageJ software. Comparisons between different groups were analyzed by unpaired student’s *t* tests, two-way ANOVA (followed by the Dunnett’s test) or log rank test using GraphPad Prism 9.5 as indicated in the figure legend. For all the above statistical analyses, significance levels are ∗*p* < 0.05, ∗∗*p* < 0.01, ∗∗∗*p* < 0.001, ∗∗∗∗*p* < 0.0001, and *p* < 0.05 or less was considered significant.

## Supplemental information

Document S1. Figures S1–S6, Tables S1, S3, S4, Data S1 and S2

Table S2. Predicted drugs from the three platforms and their intersecting candidates, related to Figure 2H
