# Supplementary material for: Sunitinib induces immunogenic cell death through eIF2α phosphorylation to potentiate immunotherapy in HCC
Source: iScience. 2026 Mar 16;29(4):115378. doi: 10.1016/j.isci.2026.115378 (PMC13092027; doi:10.1016/j.isci.2026.115378)
Supplement: Document S1. Figures S1–S6, Tables S1, S3, S4, Data S1 and S2 [file mmc1.pdf]

iScience, Volume *29*

Supplemental information

Sunitinib induces immunogenic cell death

through eIF2α phosphorylation

to potentiate immunotherapy in HCC

Yimei Gu, Hanbing Mai, Peijian Huang, Hui Yuan, Mingxuan Sun, Huixin Gao, Chenyu Shang, Yangfen Ou, Ting Liu, Xianzhang Huang, and Jizhou Tan

Supplemental Information

Supplementary Figures and legends

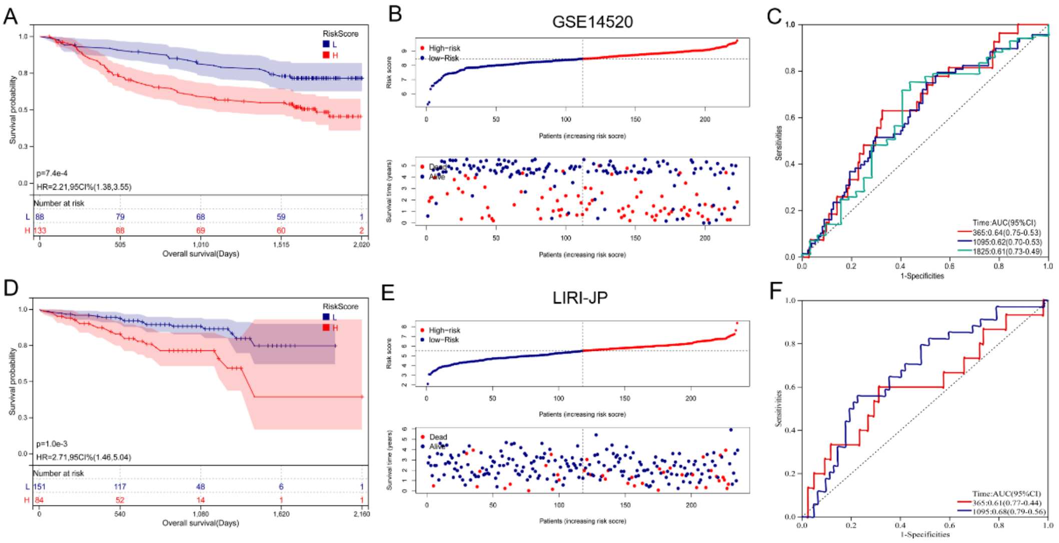

Fig. S1. Validation of the prognostic model in GSE14520 and LIRI-JP Cohort.

(A and D) Stratified Survival by Risk Groups: Kaplan-Meier curves of patients in GSE14520 (A) and LIRI-JP Cohort (D).

(B and E) Risk score and survival time distribution across patients in GSE14520 (B) and LIRI-JP Cohort (E).

(Cand F) The AUCs of the ROC curves for 1-, 3-, and 5-year OS prediction in GSE14520 (C) and LIRI-JP Cohort (F).

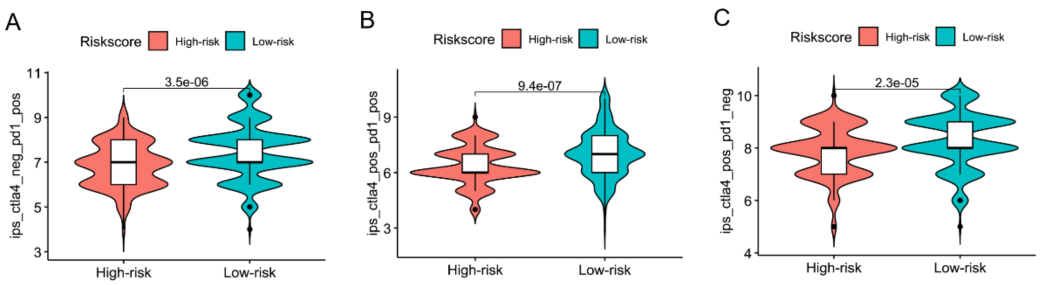

Fig. S2. The distribution patterns of immunophenotype scores (IPS) stratified by CTLA-4/PD-1 expression status — (A) $CTLA4^{-}PD1^{+}$, (B) $CTLA4^{+}PD1^{+}$, and (C) $CTLA4^{+}PD1^{-}$ — were compared between high-risk and low-risk groups in the TCGA-LIHC cohort.

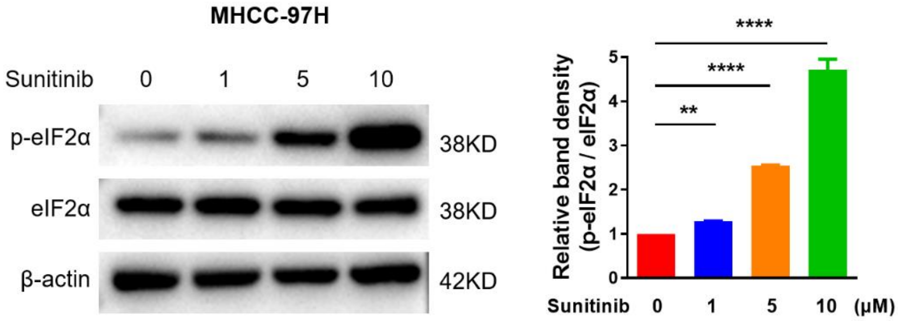

Fig. S3. Western blot analysis revealed that the treatment of sunitinib significantly increased p-eIF2α levels in MHCC-97H cells. (*n*=3).

Data are presented as means ± SD. Statistical analysis was performed using a Student's t test. **, $p < 0.05$; **, $p < 0.01$; ***, $p < 0.001$; ****, $p < 0.0001$.*

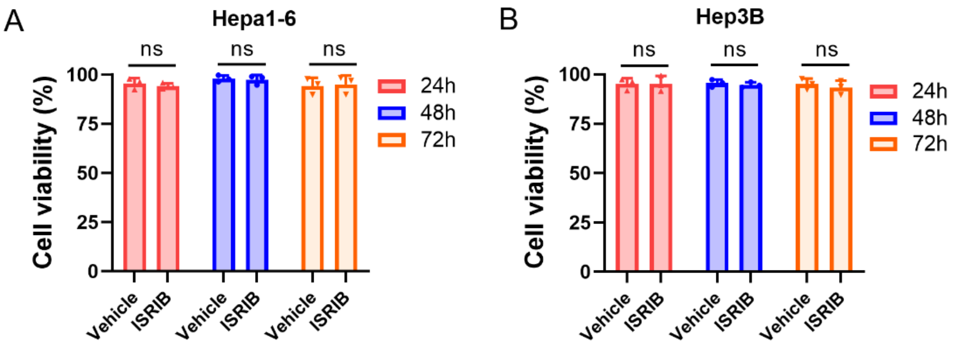

Fig. S4. Cell viability was measured by CCK-8 assay. Hepa1-6 (A) and Hep3B (B) cells were treated with ISRIB at indicated concentrations for 24, 48, or 72 hours. Data are shown as mean ± SD. (*n*=3). Statistical analysis was performed using a Student's t test. *ns*: not significant.

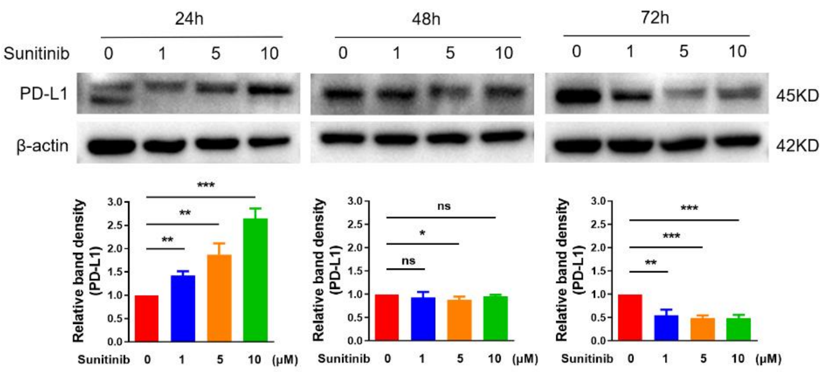

Fig. S5. Western blot analysis and quantification of PD-L1 expression in Hepa1-6 cells treated with sunitinib for 24, 48, and 72 hours.

(A) Representative Western blot images showing PD-L1 protein levels in Hepa1-6 and Hep3B cells after treatment with the indicated concentrations of sunitinib for 24, 48, and 72 hours.

(B) Densitometric quantification of PD-L1 expression normalized to β-actin at each time point. Data are presented as means ± SD. (*n*=3).

Data are presented as means ± SD. Statistical analysis was performed using a Student's t test. **, p < 0.05; **, p < 0.01; ***, p < 0.001.*

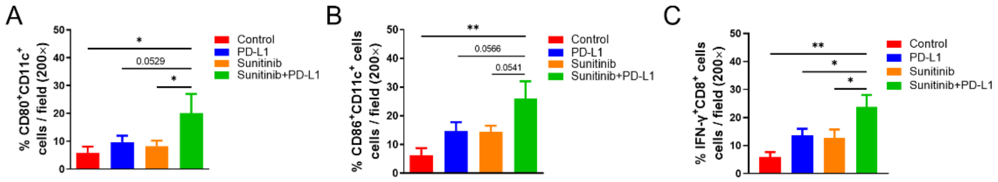

Fig. S6. The quantification of multiplex immunofluorescence staining of the markers shown in the diagram in Hepa1-6-induced subcutaneous HCC model. (*n* = 3).

Data are presented as means ± SD. Statistical analysis was performed using a Student's t test. **, p < 0.05; **, p < 0.01.*

Supplementary table S1： 52 differentially expressed genes (DEGs) of immunogenic cell death.

**No.**	**Gene**	**No.**	**Gene**	**No.**	**Gene**	**No.**	**Gene**
**1**	CD4	**14**	IL17RA	**27**	ATG7	**40**	CASP3
**2**	CCL2	**15**	CCL8	**28**	AGER	**41**	PDIA3
**3**	FPR1	**16**	EIF2A	**29**	IL2RG	**42**	TAP2
**4**	CCL4	**17**	P2RY2	**30**	SNAP25	**43**	HSP90AA1
**5**	TLR4	**18**	CTLA4	**31**	ENTPD1	**44**	CXCL10
**6**	NT5E	**19**	MLKL	**32**	STAT1	**45**	CALR
**7**	IL2RB	**20**	TLR5	**33**	SLAMF8	**46**	HSPA5
**8**	MYD88	**21**	CXCR3	**34**	TGFB1	**47**	IRF3
**9**	CD69	**22**	RIPK1	**35**	CASP8	**48**	HSPA1A
**10**	IL1B	**23**	LAMP1	**36**	ATF6	**49**	TAP1
**11**	TLR3	**24**	HMGB1	**37**	EIF2A	**50**	SQSTM1
**12**	IL1R1	**25**	PDCD1	**38**	LY96	**51**	BAX
**13**	IL6	**26**	TP53	**39**	VAMP1	**52**	CD24

Supplementary table S3：The 112 ICD-related gene set.

No.	Gene	No.	Gene	No.	Gene	No.	Gene
**1**	AGER	**29**	CGAS	**57**	IL1B	**85**	PTPN7
**2**	AIM2	**30**	CLEC9A	**58**	IL1R1	**86**	PTPRC
**3**	ANXA1	**31**	CTLA4	**59**	IL2RB	**87**	RIPK1
**4**	APOBEC3G	**32**	CXCL1	**60**	IL2RG	**88**	RIPK3
**5**	ARHGAP9	**33**	CXCL10	**61**	IL6	**89**	SAMD3
**6**	ATF6	**34**	CXCL8	**62**	IRF3	**90**	SLAMF8
**7**	ATG5	**35**	CXCR2	**63**	IRF7	**91**	SNAP25
**8**	ATG7	**36**	CXCR3	**64**	LAMP1	**92**	SQSTM1
**9**	BAX	**37**	DDX58	**65**	LCP2	**93**	STAT1
**10**	BCL2	**38**	eIF2A	**66**	LILRB1	**94**	TAGAP
**11**	CALR	**39**	EIF2AK3	**67**	LRP1	**95**	TAP1
**12**	CASP1	**40**	ENTPD1	**68**	LY96	**96**	TAP2
**13**	CASP3	**41**	FERMT3	**69**	MAP1LC3B	**97**	TFAM
**14**	CASP8	**42**	FOXP3	**70**	MAP1LC3A	**98**	TGFB1
**15**	CCL2	**43**	FPR1	**71**	IFIH1	**99**	TLR1
**16**	CCL4	**44**	GZMA	**72**	MLKL	**100**	TLR2
**17**	CCL5	**45**	HAVCR2	**73**	MYD88	**101**	TLR3
**18**	CCL8	**46**	HMGB1	**74**	NCF1	**102**	TLR4
**19**	CCR2	**47**	HSPA1A	**75**	NLRP3	**103**	TLR5
**20**	CCR5	**48**	HSP90AA1	**76**	NT5E	**104**	TLR6
**21**	CD2	**49**	HSPA5	**77**	P2RX7	**105**	TLR7
**22**	CD24	**50**	IFNAR1	**78**	P2RY2	**106**	TLR8
**23**	CD247	**51**	IFNG	**79**	PANX1	**107**	TLR9
**24**	CD4	**52**	IFNGR1	**80**	PDCD1	**108**	TNF
**25**	CD53	**53**	IL10	**81**	PDIA3	**109**	TNFRSF1A
**26**	CD69	**54**	IL12RB1	**82**	PIK3CA	**110**	TP53
**27**	CD8A	**55**	IL17RA	**83**	PRF1	**111**	VAMP1
**28**	CD8B	**56**	IL18	**84**	PSTPIP1	**112**	ZBP1

Supplementary table S4： The primary antibodies and corresponding TSA reagents of multiple immunofluorescence staining.

Primary antibodies	Cat#	Source	Corresponding TSA reagents	Cat#	Source
Rabbit anti-mouse CD8 alpha	ab209775	Abcam	iFluor® 488 tyramide	11060	aatbio
Rabbit anti-mouse IFN-γ antibody	DF6045	Affinity	Cy5 tyramide	11066	aatbio
Rabbit anti-mouse CD11c antibody	DF7585	Affinity	CY3 tyramide	11065	aatbio
			iFluor® 488 tyramide	11060	aatbio
Rabbit anti-mouse CD86 antibody	DF6332	Affinity	Cy5 tyramide	11066	aatbio
Rabbit anti-mouse CD80 antibody	DF7682	Affinity	iFluor® 488 tyramide	11060	aatbio

Data S1: Uncropped original Western blots

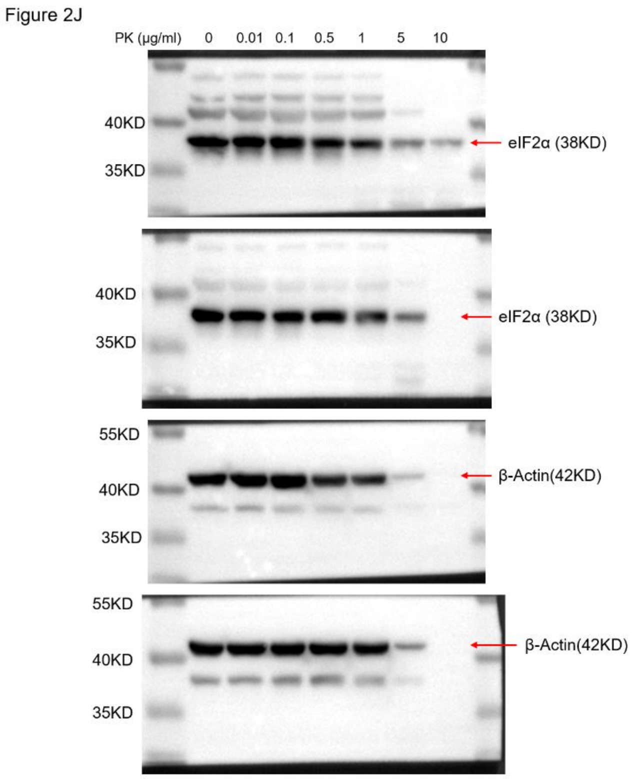

Fig. 2. (J) DARTs was performed in untreated Hepa1-6 cells lysates incubated with sunitinib at 50 μM.

Figure 3J

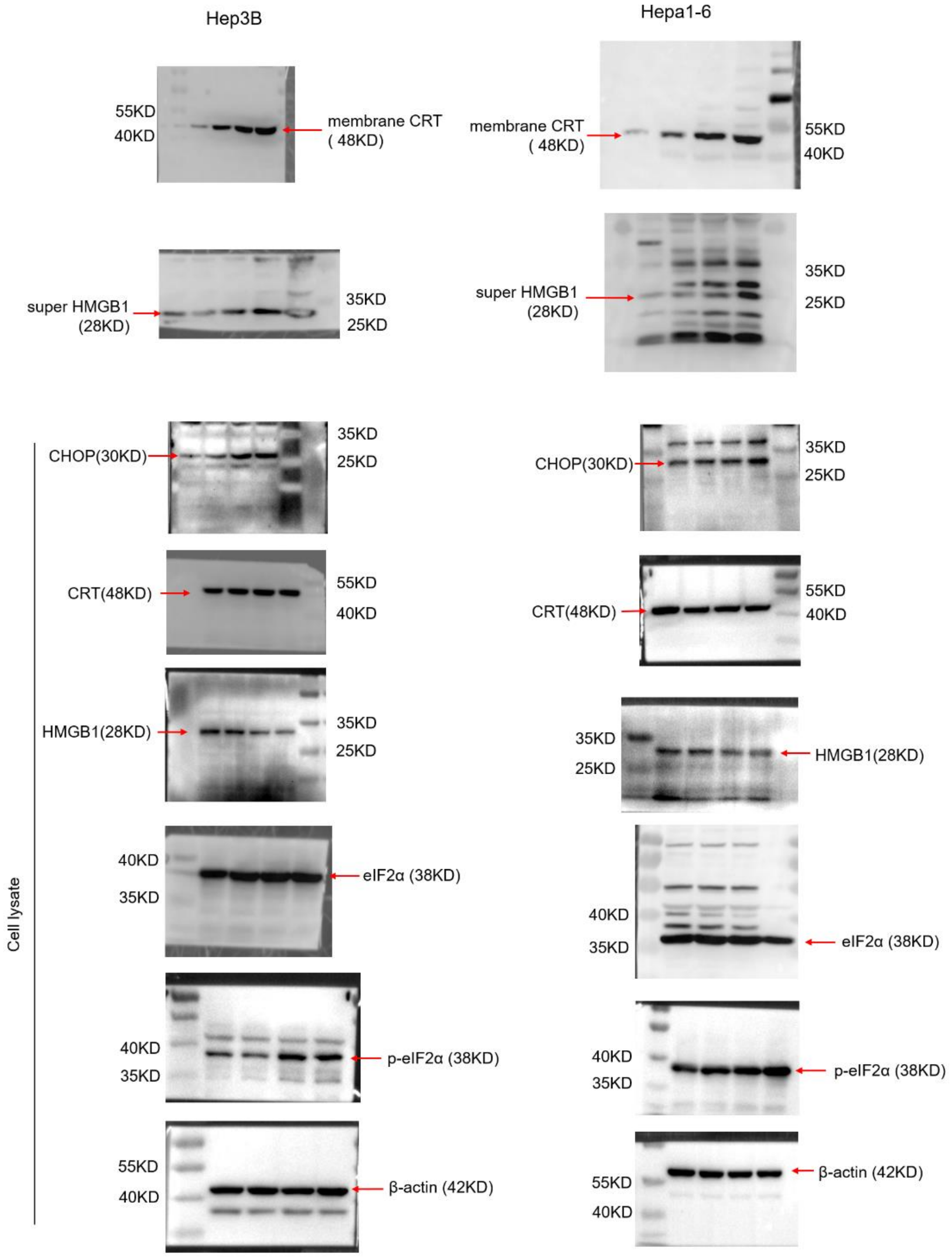

Fig. 3. (J) Signature proteins of ICD in two HCC cell lines were analyzed by Western blot assays. Cells were treated with sunitinib for 24 hours for the analysis of membrane CRT and other proteins, while HMGB1 secretion was assessed in the supernatant coll ected after 48 hours of treatment. The unlabeled CRT and HMGB1 signals originate fro m the cell membrane and the supernatant, respectively.

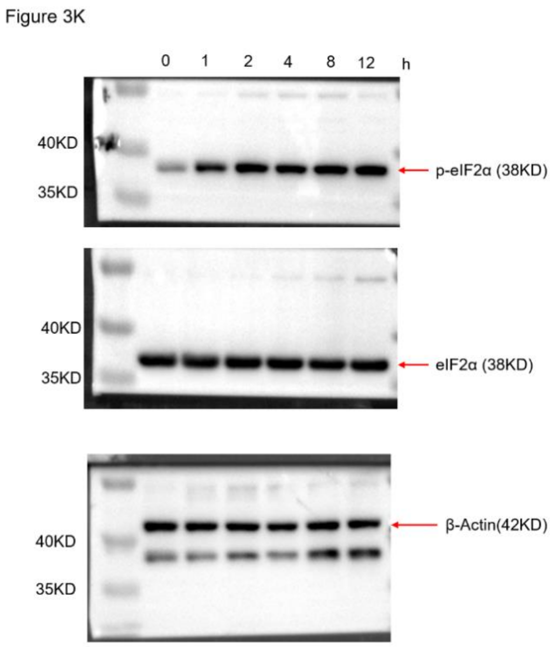

Fig. 3. (K) Immunoblots of phosphorylated eIF2α (p-eIF2α) and total eIF2α in Hepa1-6 cells treated by sunitinib for indicated time points.

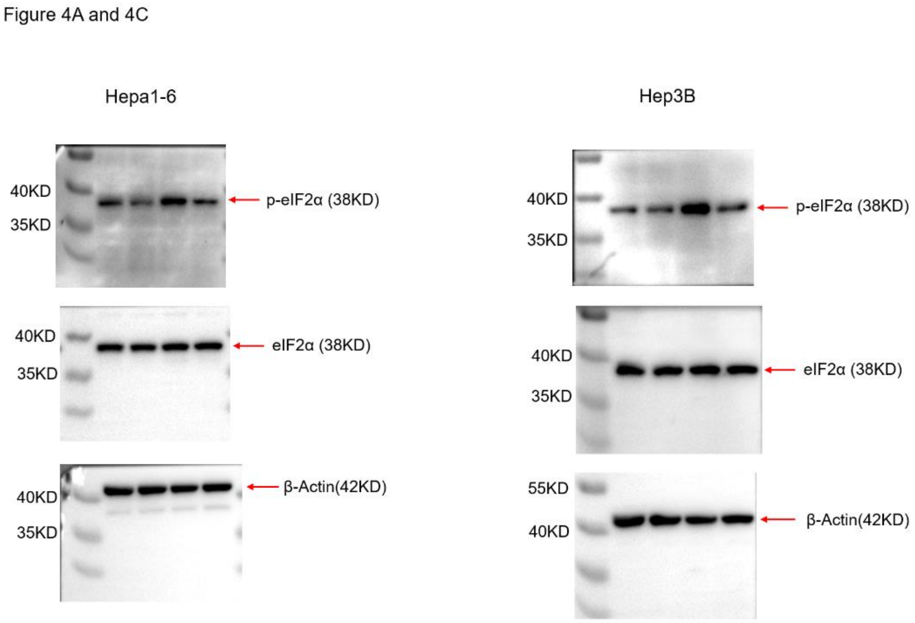

Fig. 4. (A and C) Hepa 1-6 and Hep3B cells were treated with sunitinib (5 μM) for 6 hours, with or without co-treatment with ISRIB (1 μM; added 2 hours prior to sunitinib). Protein lysates were analyzed by immunoblot for the indicated proteins.

Figure 7F

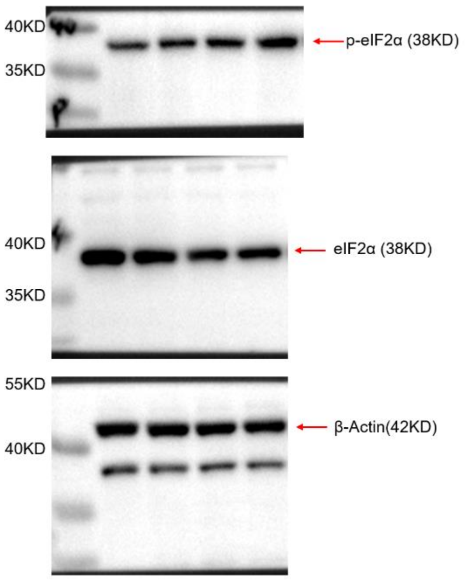

Fig. 7 (F) Western blot analysis of p-eIF2α and eIF2α protein expression levels in tumors.

Figure S3

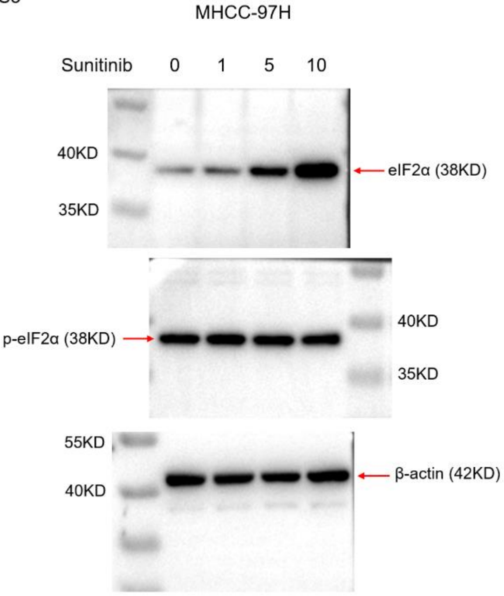

Fig. S3. Western blot analysis revealed that the treatment of sunitinib significantly increased p-eIF2α levels in MHCC-97H cells.

Figure S5

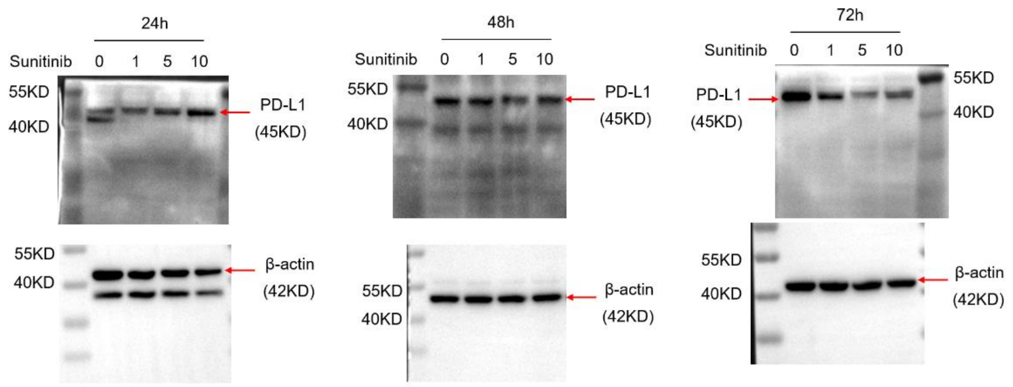

Fig. S5. Western blot analysis and quantification of PD-L1 expression in Hepa1-6 cells treated with sunitinib for 24, 48, and 72 hours.

Data S2: ARRIVE Checklist

The ARRIVE guidelines 2.0: author checklist

The ARRIVE Essential 10

These items are the basic minimum to include in a manuscript. Without this information, readers and reviewers cannot assess the reliability of the findings.

Item		Recommendation	Section/line number, or reason for not reporting
**Study design**	1	For each experiment, provide brief details of study design including:<br>a. The groups being compared, including control groups. If no control group has been used, the rationale should be stated.<br>b. The experimental unit (e.g. a single animal, litter, or cage of animals).	
**Sample size**	2	a. Specify the exact number of experimental units allocated to each group, and the total number in each experiment. Also indicate the total number of animals used.<br>b. Explain how the sample size was decided. Provide details of any *a priori* sample size calculation, if done.	
**Inclusion and exclusion criteria**	3	a. Describe any criteria used for including and excluding animals (or experimental units) during the experiment, and data points during the analysis. Specify if these criteria were established *a priori*. If no criteria were set, state this explicitly.<br>b. For each experimental group, report any animals, experimental units or data points not included in the analysis and explain why. If there were no exclusions, state so.<br>c. For each analysis, report the exact value of *n* in each experimental group.	
**Randomisation**	4	a. State whether randomisation was used to allocate experimental units to control and treatment groups. If done, provide the method used to generate the randomisation sequence.<br>b. Describe the strategy used to minimise potential confounders such as the order of treatments and measurements, or animal/cage location. If confounders were not controlled, state this explicitly.	
**Blinding**	5	Describe who was aware of the group allocation at the different stages of the experiment (during the allocation, the conduct of the experiment, the outcome assessment, and the data analysis).	
**Outcome measures**	6	a. Clearly define all outcome measures assessed (e.g. cell death, molecular markers, or behavioural changes).<br>b. For hypothesis-testing studies, specify the primary outcome measure, i.e. the outcome measure that was used to determine the sample size.	
**Statistical methods**	7	a. Provide details of the statistical methods used for each analysis, including software used.<br>b. Describe any methods used to assess whether the data met the assumptions of the statistical approach, and what was done if the assumptions were not met.	
**Experimental animals**	8	a. Provide species-appropriate details of the animals used, including species, strain and substrain, sex, age or developmental stage, and, if relevant, weight.<br>b. Provide further relevant information on the provenance of animals, health/immune status, genetic modification status, genotype, and any previous procedures.	
**Experimental procedures**	9	For each experimental group, including controls, describe the procedures in enough detail to allow others to replicate them, including:<br>a. What was done, how it was done and what was used.<br>b. When and how often.<br>c. Where (including detail of any acclimatisation periods).<br>d. Why (provide rationale for procedures).	
**Results**	10	For each experiment conducted, including independent replications, report:<br>a. Summary/descriptive statistics for each experimental group, with a measure of variability where applicable (e.g. mean and SD, or median and range).<br>b. If applicable, the effect size with a confidence interval.	

The Recommended Set

These items complement the Essential 10 and add important context to the study. Reporting the items in both sets represents best practice.

Item		Recommendation	Section/line number, or reason for not reporting
**Abstract**	11	Provide an accurate summary of the research objectives, animal species, strain and sex, key methods, principal findings, and study conclusions.	
**Background**	12	a. Include sufficient scientific background to understand the rationale and context for the study, and explain the experimental approach.<br>b. Explain how the animal species and model used address the scientific objectives and, where appropriate, the relevance to human biology.	
**Objectives**	13	Clearly describe the research question, research objectives and, where appropriate, specific hypotheses being tested.	
**Ethical statement**	14	Provide the name of the ethical review committee or equivalent that has approved the use of animals in this study, and any relevant licence or protocol numbers (if applicable). If ethical approval was not sought or granted, provide a justification.	
**Housing and husbandry**	15	Provide details of housing and husbandry conditions, including any environmental enrichment.	
**Animal care and monitoring**	16	a. Describe any interventions or steps taken in the experimental protocols to reduce pain, suffering and distress.<br>b. Report any expected or unexpected adverse events.<br>c. Describe the humane endpoints established for the study, the signs that were monitored and the frequency of monitoring. If the study did not have humane endpoints, state this.	
**Interpretation/ scientific implications**	17	a. Interpret the results, taking into account the study objectives and hypotheses, current theory and other relevant studies in the literature.<br>b. Comment on the study limitations including potential sources of bias, limitations of the animal model, and imprecision associated with the results.	
**Generalisability/ translation**	18	Comment on whether, and how, the findings of this study are likely to generalise to other species or experimental conditions, including any relevance to human biology (where appropriate).	
**Protocol registration**	19	Provide a statement indicating whether a protocol (including the research question, key design features, and analysis plan) was prepared before the study, and if and where this protocol was registered.	
**Data access**	20	Provide a statement describing if and where study data are available.	
**Declaration of interests**	21	a. Declare any potential conflicts of interest, including financial and non-financial. If none exist, this should be stated.<br>b. List all funding sources (including grant identifier) and the role of the funder(s) in the design, analysis and reporting of the study.	

ARRIVE Checklist Details

Item	Recommendation	Section/line number, or reason for not reporting
**Study design**	1b	The experimental unit was the individual mouse. Each mouse was independently assigned to a treatment group, received treatment, and had tumor volume and survival monitored as an independent data point.
**Sample size**	2b	The sample size for each group was determined based on logistical considerations and is consistent with established sample sizes in comparable preclinical HCC models evaluating sunitinib and immune checkpoint inhibitors *[18]*.<br>*[18] G. Li, D. Liu, T.K. Cooper, et al., Successful chemoimmunotherapy against hepatocellular cancer in a novel murine model, Journal of hepatology, 66 (2017) 75-85.*
**Randomisation**	4b	To minimise potential confounders:<br>• Treatment order: All treatments (oral gavage, intraperitoneal injection) were administered in an order that was varied daily to avoid time-of-day effects.<br>• Outcome assessment: Measurement of tumor volume was performed by an investigator who was blinded to the group allocations.<br>• Animal/Cage location: Cage location in the animal facility was not systematically rotated as a controlled variable. This was not considered a major confounder under the standard housing conditions.
**Blinding**	5	• During allocation: The investigator performing randomisation was aware.<br>• During the conduct: Personnel administering treatments were aware.<br>• During the outcome assessment: The investigator measuring tumor volume was blinded.<br>• During the data analysis: Researchers were not blinded to group allocation during data analysis.

Item	Recommendation	Section/line number, or reason for not reporting
**Outcome measures**	6b	This is primarily a hypothesis-generating discovery study. The sample size was chosen based on logistical considerations and consistency with established models in the field *[18]*, which are sufficiently powered to detect the large effect sizes expected in these pre-clinical models.<br>*[18] G. Li, D. Liu, T.K. Cooper, et al., Successful chemoimmunotherapy against hepatocellular cancer in a novel murine model, Journal of hepatology, 66 (2017) 75-85.*
**Statistical methods**	7b	The assumption of normality for parametric tests (t-test, ANOVA) was assessed using the Shapiro-Wilk test. For data that did not meet the normality assumption, equivalent non-parametric tests were employed. The assumption of equal variance for ANOVA was verified using Brown-Forsythe test.
**Results**	10b	Effect sizes with confidence intervals are not routinely reported for all experiments in this study. However, the primary data is available to calculate effect sizes if required.
**Housing and husbandry**	15	Mice in this study were housed under specific pathogen-free (SPF) conditions in the Guangzhou University of Chinese Medicine Animal Facility. Animals were maintained in a controlled environment (12-h light/dark cycle, 22 ± 1°C, 55 ± 10% humidity) and group-housed (4-5 mice per cage) in individually ventilated cages (390×195×180mm) with corncob bedding, nesting material. Food and water were provided *ad libitum*. All procedures were approved by the Welfare and Ethical Committee for Experimental Animal Care of Guangzhou University of Chinese Medicine (Approval No. 20240417001).
**Protocol registration**	19	No formal study protocol was preregistered. However key design features such as sample size randomization and primary outcomes were determined beforehand using pilot studies and power calculations. The main analytical approach was also predefined.
